# Multiple Functional Brain Networks Related to Pain Perception Revealed by fMRI

**DOI:** 10.1007/s12021-021-09527-6

**Published:** 2021-06-08

**Authors:** Matteo Damascelli, Todd S. Woodward, Nicole Sanford, Hafsa B. Zahid, Ryan Lim, Alexander Scott, John K. Kramer

**Affiliations:** 1 Department of Psychology, University of British Columbia, 2136 West Mall, Vancouver, BC V6T 1Z4 Canada; 2 BC Mental Health & Addictions Research Institute, BC Children’s Hospital Research Institute, 938 West 28th Ave, Vancouver, BC V5Z 4H4 Canada; 3 ICORD, Blusson Spinal Cord Centre, 818 West 10th Ave, Vancouver, BC V5Z 1M9 Canada; 4 Department of Psychiatry, University of British Columbia, 2255 Wesbrook Mall, Vancouver, BC V6T 2A1 Canada; 5 Department of Physical Therapy, University of British Columbia, 2177 Wesbrook Mall, Vancouver, BC V6T 1Z3 Canada; 6 Centre for Hip Health and Mobility, Robert H. N. Ho Research Centre, 2635 Laurel St, Vancouver, BC V5Z 1M9 Canada; 7 School of Kinesiology, University of British Columbia, 6081 University Blvd, Vancouver, BC V6T 1Z1 Canada

**Keywords:** Functional MRI, Functional brain networks, Functional connectivity, Pain, Multivariate least-squares regression, Principal component analysis, Hemodynamic responses, Attention

## Abstract

The rise of functional magnetic resonance imaging (fMRI) has led to a deeper understanding of cortical processing of pain. Central to these advances has been the identification and analysis of “functional networks”, often derived from groups of pre-selected pain regions. In this study our main objective was to identify functional brain networks related to pain perception by examining whole-brain activation, avoiding the need for a priori selection of regions. We applied a data-driven technique—Constrained Principal Component Analysis for fMRI (fMRI-CPCA)—that identifies networks without assuming their anatomical or temporal properties. Open-source fMRI data collected during a thermal pain task (33 healthy participants) were subjected to fMRI-CPCA for network extraction, and networks were associated with pain perception by modelling subjective pain ratings as a function of network activation intensities. Three functional networks emerged: a sensorimotor response network, a salience-mediated attention network, and the default-mode network. Together, these networks constituted a brain state that explained variability in pain perception, both within and between individuals, demonstrating the potential of data-driven, whole-brain functional network techniques for the analysis of pain imaging data.

**Supplementary Information:**

The online version contains supplementary material available at 10.1007/s12021-021-09527-6.

## Introduction

The application of non-invasive neuroimaging techniques has greatly enhanced our neurobiological understanding of pain (Davis, 2011; May, 2008; Moayedi et al., 2018). Functional magnetic resonance imaging (fMRI) has played a particularly valuable role, leading to the discovery of a core set of regions—including the thalamus, the anterior cingulate, somatosensory, and insular cortices—that are consistently activated by experimental pain (Davis & Moayedi, 2013; Iannetti & Mouraux, 2010; Mouraux & Iannetti, 2018; Wilcox et al., 2015).

Traditionally, fMRI research on pain has relied extensively on mass-univariate analysis techniques to investigate the functional role of individual regions in generating the pain experience. More recently, functional connectivity (FC) techniques, which examine temporal correlations between regions, have allowed researchers to determine how traditional pain regions organize into larger networks. Characterizing such networks (in terms of both spatial organization and function) is an important objective because (1) largely distributed patterns of activation likely provide a more reliable “signature” of pain than any local activation, where signatures have the potential to be used in diagnosis and/or evaluations of treatment efficacy (van der Miesen et al., 2019), and (2) understanding network functionality informs our basic understanding of existing treatments, for example, cognitive-behavioural therapies (Eccleston et al., 2013), as well as burgeoning treatment avenues like neuromodulation (Alo & Holsheimer, 2002) and real-time fMRI feedback (Chapin et al., 2012).

In pain research, FC techniques have shown traditional pain regions to be organized into distinct functional networks serving sensory, emotional, cognitive or motor aspects of pain (Wilcox et al., 2015). However, FC studies have often relied on seed-based techniques, meaning that correlations between brain regions are interrogated by selecting a voxel or region (a “seed”) and modeling activity in other voxels as a function of signal changes within the seed (Diano et al., 2016; Moayedi et al., 2018; Wilcox et al., 2015). Estimated model parameters represent the strength of each voxel’s functional connection to the seed and can be used to construct a map of intercorrelated regions, that is to say, a functional network (Moayedi et al., 2018). Although powerful, this framework is limited by the regions (or seeds) inputted as regressors. It is therefore important to consider alternative methods that are data-driven, letting functional networks emerge without relying on spatial (i.e. regions-of-interest) or temporal assumptions (i.e. pre-supposing the *shape* of the response elicited, as is typically done in the univariate framework; Henson & Friston, 2007).

One such alternative is Constrained Principal Component Analysis for fMRI (fMRI-CPCA). fMRI-CPCA extracts functional brain networks from whole-brain Blood Oxygen Level Dependent (BOLD) signal data with variance constrained to that predictable from task timing, and generates spatial maps, as well as estimates of hemodynamic responses (HDRs) for each combination of subject, task condition and brain network. The technique is valuable in that it combines: (1) networks based on multivariate analyses, which interrogate the intercorrelated structure of task-based voxel data without submitting each voxel to a separate statistical test as in univariate approaches (e.g. seed-based connectivity techniques, where each voxel is correlated to the seed), (2) networks extracted from BOLD signal constrained to task-timing-related variance, which is useful because task-optimized networks can be more readily associated with cognitive and behavioural functions by analysing how network HDRs differ between task conditions, and (3) data-driven network extraction, meaning that no assumptions about the spatial or temporal properties of networks are formally defined. Spatial and temporal assumptions are avoided by analyzing all voxels in the brain instead of selecting regions-of-interest and using a Finite Impulse Response (FIR) model of task-evoked HDRs instead of assuming a particular HDR shape, respectively.

In this paper, we used fMRI-CPCA to conduct a whole-brain, data-driven extraction of functional networks involved in pain. We analyzed a publicly available and previously published dataset, posted on openneuro.org (accession number ds000140; Gorgolewski, 2018; Woo et al., 2015), featuring a thermal stimulation task. fMRI-CPCA delineated multiple, dominant functional brain networks evoked by thermal stimulation, obtaining estimates of their spatial configurations and temporal response patterns. We then modelled subjective pain ratings as a linear function of multiple network activations, to verify the relevance of the networks detected to pain perception. Our fundamental goal was to identify the functional networks involved in processing noxious heat stimuli, explore their responses and anatomy, and quantify their relationships with pain perception. Based on research that has demonstrated the organization of pain regions into distinct networks at rest (described as sensory-discriminative, cognitive-evaluative, affective-motivational, and motor networks; Davis & Moayedi, 2013; Wilcox et al., 2015), we hypothesized similar network configurations to be evoked during experimental thermal pain based on our fMRI-CPCA analysis.

## Materials and Methods

The original study by Woo et al. (2015) provides detailed information on participants, study design and data collection. Here, we provide only a brief description for clarity.

### Participants

33 healthy, right-handed adults (22 females, 11 males) participated in the study, with a mean age of 27.9 years (SD = 9.0 years). All participants provided informed consent and reported no prior history of psychiatric, neurological or pain disorders. Ethical review and approval were provided by the Columbia University Institutional Review Board (Protocol number AAAE3743). Since the data were anonymized and we performed a secondary analysis, no local ethics review was required.

In our study, two participants (subjects 11 and 30) were excluded because they received too few trials under each experimental condition (defined below), creating problems for the fMRI-CPCA algorithm. This left 31 participants to be analysed.

### Thermal Stimulation

To elicit pain, a thermode device was placed on the volar surface of the left forearm (TSA-II Neurosensory Analyzer with a 16-mm Peltier thermode endplate, Medoc Advanced Medical Systems). Thermal stimuli were delivered at specific temperatures for 12.5 s each, with 3 s of ramp-up, 7.5 s at the target temperature, and 2 s of ramp-down. Temperature levels ranged from 40.8 °C to 47.3 °C (study documentation and participant results are available at https://openneuro.org/datasets/ds000140/versions/00001).

### fMRI Task

Participants completed 9 separate functional scanning sessions. There were 3 types of sessions: “standard” runs, where pain stimulation was received passively; “regulate-up” runs, where participants were instructed to increase the intensity of pain by cognitive control; and their counterpart, “regulate-down” runs. The regulation manipulation was intended to engage supplementary brain systems for pain regulation. For the explicit purposes of our study, we focused on standard runs only.

**Fig. 1:**
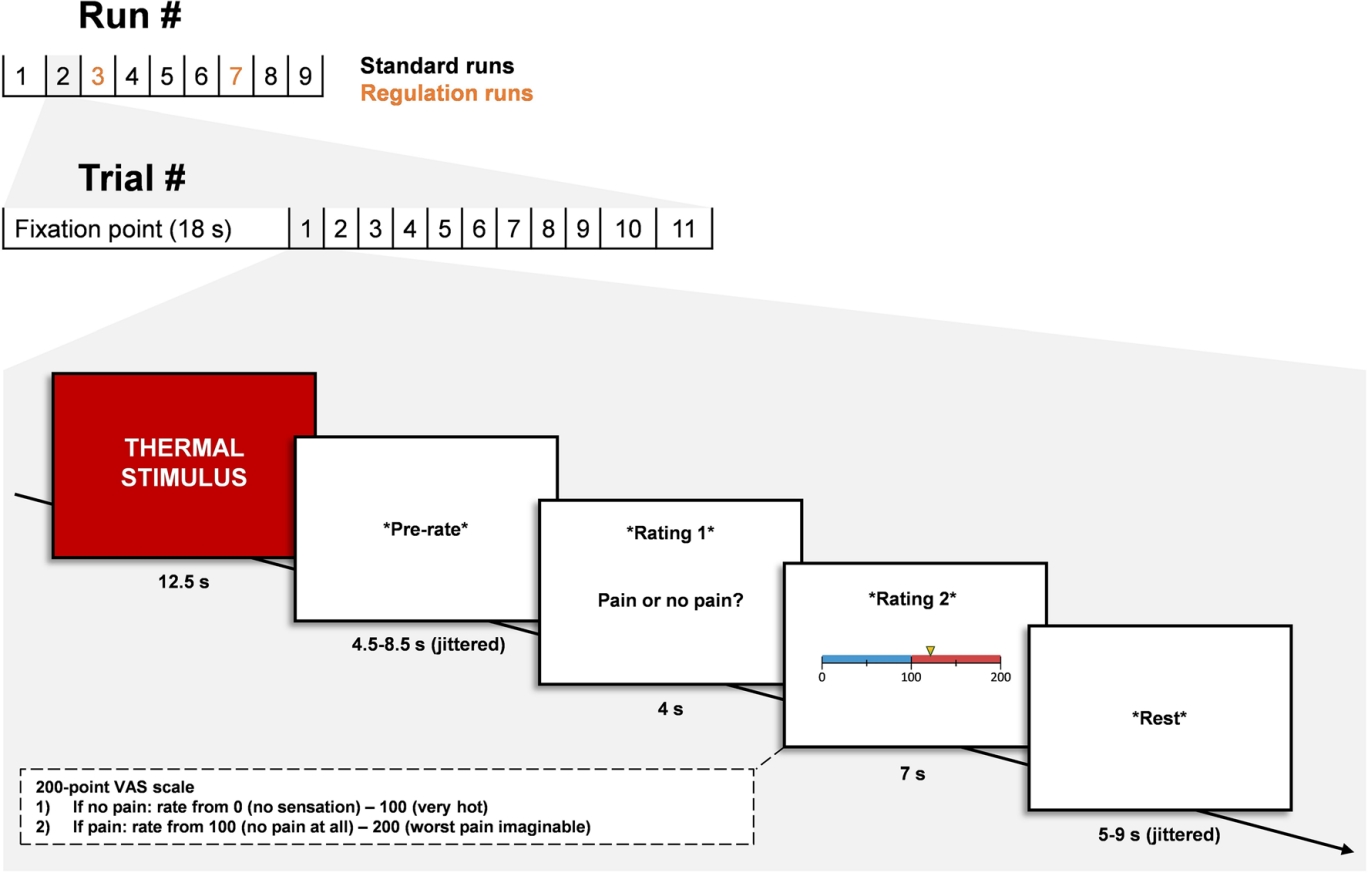
Schematic illustration of task design adapted from Woo et al. (2015). Every run was preceded by an 18 s fixation cross presented on screen. Every trial began with a 12.5 s thermal stimulus, followed by a pre-rate anticipation period (4.5–8.5 s, jittered), a 4 s rating period to judge if the stimulus was painful or not, a 7 s pain rating period using a VAS scale, and a post rate rest period of 5–9 s (jittered)

Each standard run began with an 18-s fixation cross presented on screen, followed by 11 consecutive trials. Each trial was 33–41 s long and featured the same progression: 12.5 s of thermal stimulation, 4.5–8.5 s (jittered) of pre-rate rest, 11 s of pain rating (completed on screen using a hand-held remote), and 5–9 s (jittered) of post-rate rest. The rating period involved two kinds of rating; first, participants decided whether a stimulus was painful or not (this phase lasted 4 s), then participants rated the intensity of their sensation on a Visual Analogue Scale from 0 to 200, where the interval 0–100 represented non-painful warmth, and 100–200 represented the intensity of a stimulus perceived as painful. The scale was presented on screen, and participants were instructed as to the meaning of each interval prior to scanning. The specific order of temperatures administered throughout each run can be found in Woo et al. (2015). For a schematic illustration of task design, see Fig. 1.

### Image Acquisition

Whole-brain functional images were collected on a 3 T Philips Achieva TX scanner at Columbia University’s Program for Imaging in Cognitive Science (PICS). Structural images were collected with high-resolution T1 spoiled gradient recall images (SPGR), which allow for anatomical localization and warping to standard space. For functional EPI image collection, the following scanning parameters were set: TR  =  2000 ms, TE  =  20 ms, field of view  =  224 mm, 64 × 64 matrix, 3 × 3 × 3 mm^3^ voxels, 42 interleaved slices, parallel imaging, SENSE factor 1.5. E-Prime software (PST Inc.) was used to control stimulus presentation and collect behavioural data.

### Preprocessing

For our analysis, all preprocessing was completed in SPM 12. Structural and functional scans were reoriented manually, such that the origin was placed on the anterior commissure, and the AC-PC plane was oriented horizontally. Slice-time correction was performed to mitigate the temporal lag in slice acquisition across the 2-s TR, using slice 21 as a reference. Realignment algorithms were applied to counteract displacement of voxels due to head movement, and runs that exceeded movement parameter thresholds of 4.5 mm in either z, x, y direction, as well as pitch, yaw or roll, by at least 50 scans, were removed from the analysis (subject 10, run 5 and 6; subject 2, run 5; subject 4, run 1, 4 and 5). For each participant, functional scans were co-registered to their corresponding structural images, and structural T1 scans were segmented into gray matter, white matter, cerebrospinal fluid, meninges and skull components. Finally, raw functional data were normalized to MNI template space (with a voxel size of 3 × 3 × 3 mm) and smoothed with a 6 × 6 × 6 FWHM Gaussian kernel.

For detailed explanations of preprocessing methods, along with specific versions of software tools used, refer to supplementary materials.

### Task-Based Whole-Brain Network Analysis

Constrained Principal Component Analysis (CPCA) is a statistical technique that combines multivariate least-squares regression with principal component analysis (Hunter & Takane, 2002; Takane & Shibayama, 1991; Takane & Hunter, 2001). It can be used to perform whole-brain analyses of fMRI BOLD signal data. When applied to fMRI, it identifies multiple functional networks involved in a task and estimates fluctuations in BOLD signal for each network, over a specified interval of time. Further statistical tests can be used to quantify the interactions between networks, correlational relationships between network activation and behavioural measures, and the effect of experimental manipulations on the activation of each network.

Broadly speaking, fMRI-CPCA involves two steps. First, multivariate least-squares multiple regression is used to isolate variance in BOLD signal that is predictable from the timing of stimulus presentation, after which the variance is said to be “constrained” to task timing. This first step is referred to as the external analysis. Second, a principal component analysis (PCA) is conducted on the constrained portion of the variance in BOLD signal, and the extracted components represent systems of functionally interconnected voxels (i.e. functional brain networks) related to the task. This step is referred as the internal analysis. Importantly, applying PCA *after* the regression ensures that the networks identified are based on task-related information only. This is a defining feature of fMRI-CPCA and distinguishes it from other applications of PCA (or ICA) used in fMRI. In fMRI-CPCA, the variance shared between principal components and task timing is maximized, thus avoiding any contamination of the solution by variability that is not predictable from task timing. Ultimately, fMRI-CPCA outputs brain activity maps that can be overlaid on a structural image (for example, in applications like MRIcron [https://www.nitrc.org/projects/mricron]), as well as estimated hemodynamic response shapes (plotted over post-stimulus time) for each combination of network, subject and task condition. The next few paragraphs will elaborate on specific matrices and equations required to implement the analyses.

In order to perform the external analysis, two matrices must first be prepared. The *Z* matrix (or activation matrix) contains the BOLD data for all runs, with each voxel represented as a single column, and each full-brain scan represented as a single row. In the current study, 31 subjects went through nine runs each, with 209 scans per run. Six runs were removed due to excessive head movement (see section “2.5. Preprocessing”), leaving a total of 42,427 rows (full brain scans) and 79,522 columns (voxels) in the *Z* matrix. The mean value for each voxel was centered to zero for each run separately, and the variables standardized (such that the standard deviations were set to one for each run separately). The *G* matrix (or design matrix) contains a Finite Impulse Response (FIR) model of the BOLD signal based on stimulus presentation timing; unlike more conventional models, the FIR model does not impose a predetermined HDR shape on the dataset (which is commonly assumed to aid in determining task-relevant activations in BOLD signal). Instead, a value of one is placed into cells of *G* for which the BOLD signal is to be estimated, and a value of zero is in all other cells—thus, the *G* matrix simply defines the time intervals during which we expect to see task-relevant activations. The number of rows in the *G* matrix will equal the number of rows in the *Z* matrix, but the number of columns is equal to the number of post-stimulus time points (time bins) for which the BOLD signal is to be predicted, multiplied by the total number of conditions and the total number of subjects. The *G* matrix is also standardized for each individual run. We then regress the *Z* matrix onto the *G* matrix,

$$ Z= GC+E, $$

where *C* = (*G* ′ *G*)^−1^*G* ′ *Z* is a matrix of timepoint- and voxel-specific regression weights that satisfy the least-squares criterion. When *C* is applied to *G* it provides a matrix of BOLD signal values predicted from task-timing, _scans_ $\hat{Z}$_voxels_ or *GC*. *E* represents the residual signal (i.e. signal that is not predictable from task-timing), which is disregarded in the rest of the analysis. As an additional note, *E* can be further analysed exactly like *GC*; such an analysis would produce the dominant networks that are not predictable from task timing, which may be those engaged during off-task periods, or task on processes that span the whole series of trials but are not specifically elicited by the onset of tasks. This type of analysis was not carried out here and is beyond the scope of this paper.

The next stage of fMRI-CPCA is the internal analysis, which typically involves application of a principal component analysis (PCA) to the constrained, task-related signal (*GC*). This identifies correlated structure underlying the voxel data, grouping correlated voxels into components that represent functional brain networks. Importantly, these components will be optimized to be task-related, because *GC* contains task-related variance only. PCA is achieved through singular value decomposition of *GC*:

$$ UDV^{\prime }= SVD(GC) $$

where *U* is a matrix of left singular vectors, *D* is a diagonal matrix of singular values, and *V*′ is a matrix of right singular vectors. In matrix *U*, columns represent components, and rows represent scans. The values in matrix *U* are “component scores” and provide an indication or “score” of how important each component is for each scan. In matrix *V*, columns represent components, and rows represent voxels. Cells of *V* can be rescaled by $VD/\sqrt{N}$ to obtain “component loadings”—correlation coefficients indicating the correlation of task-related BOLD signal in each voxel with the respective component scores. Voxels that are highly correlated with a given component’s “component scores” form the brain regions that define the functional network represented by that component. Notably, rescaling right singular vectors in *V* allows them to be interpreted as correlations between voxels and networks, while also providing a better approximation of the inputted matrix *GC* by incorporating the variance accounted for by each network. To visualize a brain activity map for each network, columns in rescaled *V* were overlaid on a brain template in MRIcron and thresholded to display only the voxels with the most dominant loadings (e.g. top 10% absolute values). In the current study, we orthogonally rotated and rescaled the *V* matrix prior to display, using a varimax solution with 500 iterations (Abdi & Williams, 2010; Bryant & Yarnold, 1995; Kaiser, 1958).

PCA identifies a large number of components, but a select few can be extracted (the components that account for the least amount of variability are considered noise, or brain activity that is unlikely to be reliable). Various methods for component selection exist; in this study we used the elbow method. This method relies on visual inspection of the scree plot of singular values (Cattell, 1966; Cattell & Vogelmann, 1977). When plotted, singular values (which are contained in *D*) produce a line that gradually approaches zero as components account for less and less variance. In the elbow method, components are selected for extraction by locating the first abrupt increase in variance—relative to the baseline variance accounted for by the majority of components—and extracting the associated component followed by all components that account for a greater proportion of variance (Kodinariya & Makwana, 2013). In this study, 4 components were retained and varimax rotated. We attempted additional analyses with a greater number of components retained, to ensure the validity of the chosen threshold. Additional networks did not substantially improve the solution, only marginally increasing variance explained and failing to detect new and informative regions/networks, instead fragmenting networks previously identified in the four-component solution.

After the external and internal analyses are complete, a final step is applied to produce estimates of HDR shapes associated with each network. This is achieved by relating component scores (in matrix *U*) back to stimulus presentation timing (coded in *G*), and computing *P* such that:

$$ U= GP, $$

where *P* contains “predictor weights”—these are weights that estimate the intensity of each component for the time bins specified in *G*. When plotted over post-stimulus time, predictor weights reveal the unique HDR shapes elicited by each subject and condition within each network, for the specified interval of time. In this study, predictor weights were averaged over subjects before plotting. Further, predictor weights were averaged over post-stimulus time to compute overall intensity values for network activation; more detail on this is provided below.

### Preparation of *G*

The goal of the current study was to determine how the brain configures itself when processing pain, and to use the brain networks detected to generate a model of subjective pain perception. Accordingly, we formatted the *G* matrix such that separate HDR shapes would be produced for high and low temperature conditions. The division was based on the median temperature administered across all trials, including regulation runs (the median temperature was 44.3 °C, any stimulus that was equal to or less than 44.3 was assigned to the “low” temperature condition; the rest were “high”). We examined brain activity during thermal heat portions of the experimental task only, and only included standard runs in the analysis to avoid capturing brain systems for cognitive self-regulation over pain perception. The task-relevant time interval (encoded in *G*) was defined as the 16 s immediately following thermal stimulus presentation; in this way, the entire duration of the stimulus and 3.5 s thereafter were accounted for. Because each full-brain fMRI scan was completed in two seconds, HDRs were estimated for eight post-stimulus time bins. The *G* matrix therefore consisted of 496 columns (2 conditions × 31 subjects × 8 time bins), and 42,427 rows (equal to the number of rows in the *Z* matrix).

### Preparation of Network and Rating Data for Multiple Regressions

For each network, the predictor weights produced in the final step of fMRI-CPCA define the unique HDR shapes associated with each condition, over the specified 16-s time interval. To model pain perception from brain activation, it was preferable to compute a single value that would capture the intensity of the response. In this case, due to exploration of the HDR shapes obtained, network activation intensity was estimated by averaging predictor weights (i.e. estimated BOLD signal) over the entire post-stimulus time interval. This yielded 248 estimates in total, one for each temperature category for each of the four networks detected by fMRI-CPCA for every participant.

Pain ratings were subjected to a similar procedure: ratings associated with each temperature condition were averaged over trials to obtain participant-specific estimates of pain perception during high- and low-temperature stimuli, yielding 62 estimates in total.

### Multiple Regressions

Two separate multiple linear regression analyses were conducted on pain rating and network activation data.

#### Modelling Within-Subject Pain

The first of these modelled *changes* in perceived pain as a function of changes in the intensity of network activations. The fundamental goal here was to examine how a change in rating corresponded with changes in activation intensities between high and low temperatures. All brain networks detected by fMRI-CPCA (component 3 was excluded because it reflected a movement artifact, see section "3. Results") were inputted as predictors to explain changes in pain rating:

$$ \Delta \mathrm{Rating}\sim 1+\Delta \mathrm{Component}1+\Delta \mathrm{Component}2+\Delta \mathrm{Component}4. $$

To evaluate model fit beyond *R*^*2*^, fitted values were plotted against, and correlated with, the response variable using Pearson’s r correlation coefficient.

#### Modelling Between-Subject Pain

To investigate the relationship between perceived pain and network activation intensity across subjects, we applied a bootstrap-like regression procedure. Samples of size *n* = 31 were drawn from the dataset of condition- and participant-specific estimates of pain ratings and network activation intensities (see section “2.8. Preparation of Network and Rating Data for Multiple Regressions”). Each participant contributed one pain rating (and its corresponding network intensities), selected at random, and every sample was a near-balanced combination of ratings greater than 100 (i.e. painful) and ratings less than 100 (i.e. warm). The prevalence of pain in the sample was maintained between 45% and 55% (non-inclusive).

All brain networks detected by fMRI-CPCA (components 1, 2, and 4) were then used to model the pain rating variable,

$$ \mathrm{Rating}\sim 1+\mathrm{Component}1+\mathrm{Component}2+\mathrm{Component}4, $$

for every sample drawn (305 samples in total). This effectively treated the regressors as random rather than fixed effects (Fox, 2015), and it provided empirical bootstrap distributions for relevant statistics like regression coefficients, *R*^*2*^ and model significance as determined by F-test, from which estimates of each metric or model parameter could be obtained. Confidence intervals (95%) were calculated non-parametrically using percentiles (Fox, 2015).

Model fit was further evaluated by determining the accuracy with which fitted values distinguished between pain and warmth. This was done by converting the ratings predicted by the model (a continuous variable) into a categorical outcome, pain or non-pain, based on the 100-point pain threshold specified by the VAS scale. For every model, the predicted binary outcomes were compared to the true state of affairs in order to generate estimates of accuracy (the proportion of total cases that were correctly classified as either pain or warmth), sensitivity (the proportion of pain cases that were correctly classified as pain) and specificity (the proportion of non-pain cases that were correctly classified as warmth). This provided empirical bootstrap distributions and corresponding estimates (with percentile intervals) of model accuracy, sensitivity and specificity. All regression analyses were completed in MATLAB R2019a (scripts available from https://github.com/MatteoDamascelli/Multiple-Functional-Brain-Networks-Related-to-Pain-Perception-Revealed-by-fMRI.).

## Results

### Summary of fMRI-CPCA Output

**Fig. 2:**
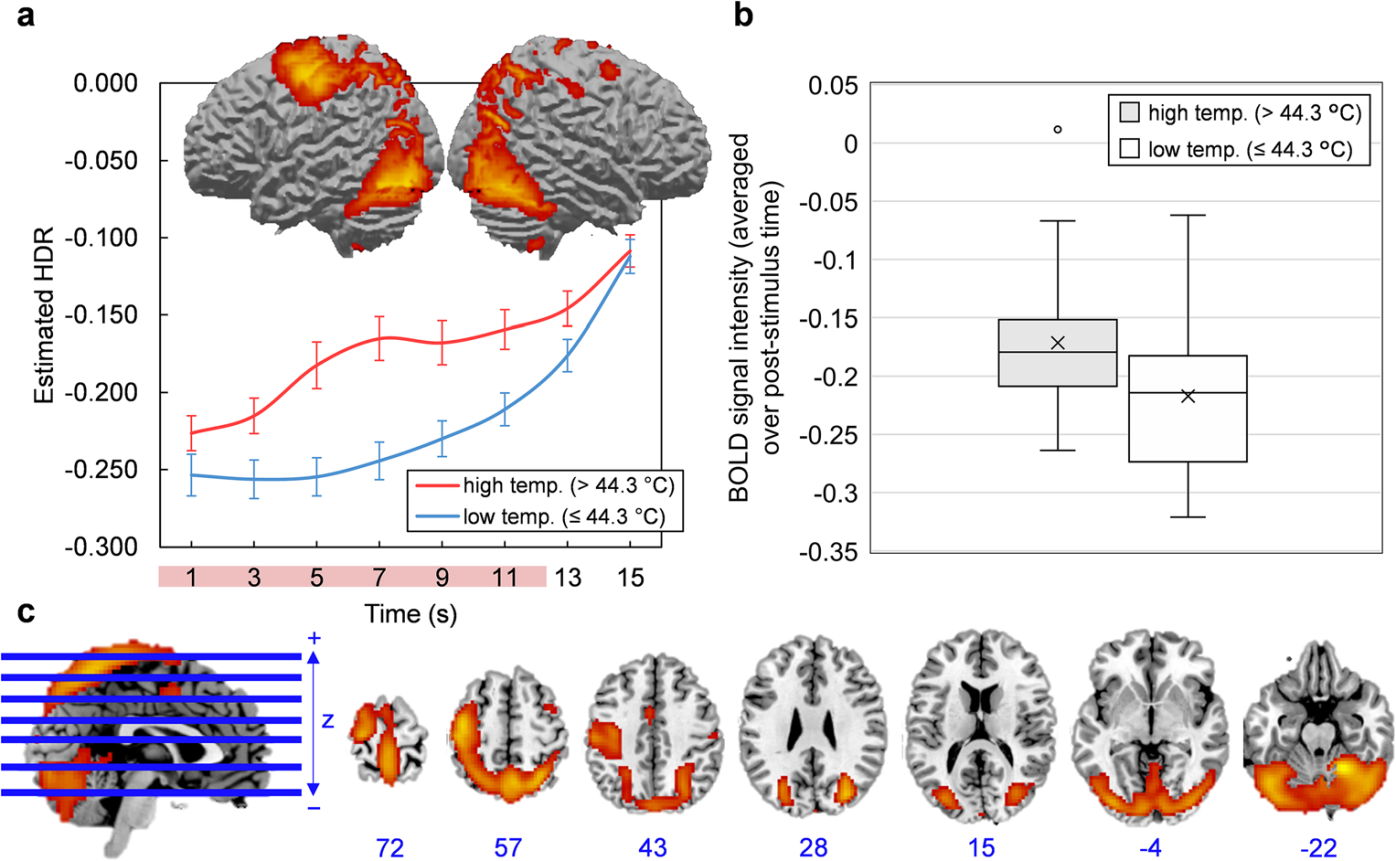
**a-c** Component 1. **a** Three-dimensional rendering of Component 1 (based on the top 10% of component loadings) and estimated HDRs associated with this network over the course of one thermal stimulation trial. The red bar placed over x-axis tick labels indicates the duration of a thermal stimulus (12.5 s). Estimated HDRs were obtained by averaging the FIR-based predictor weights for each condition level and plotting them as a function of post-stimulus time. Error bars given by standard error. HDR = hemodynamic response. **b** Boxplots illustrating the distributions of Component 1 BOLD signal across participants, for both high and low temperature stimuli. BOLD signal was first averaged over the entire post-stimulus time interval for each participant and each condition. The mean is given by ×. **c** Horizontal cross-sections of Component 1 (only the top 10% of component loadings are shown). Positive loadings in red, threshold = 0.17, max = 0.33. No negative loadings. Blue values indicate the MNI coordinate of each slice in the z direction

**Fig. 3:**
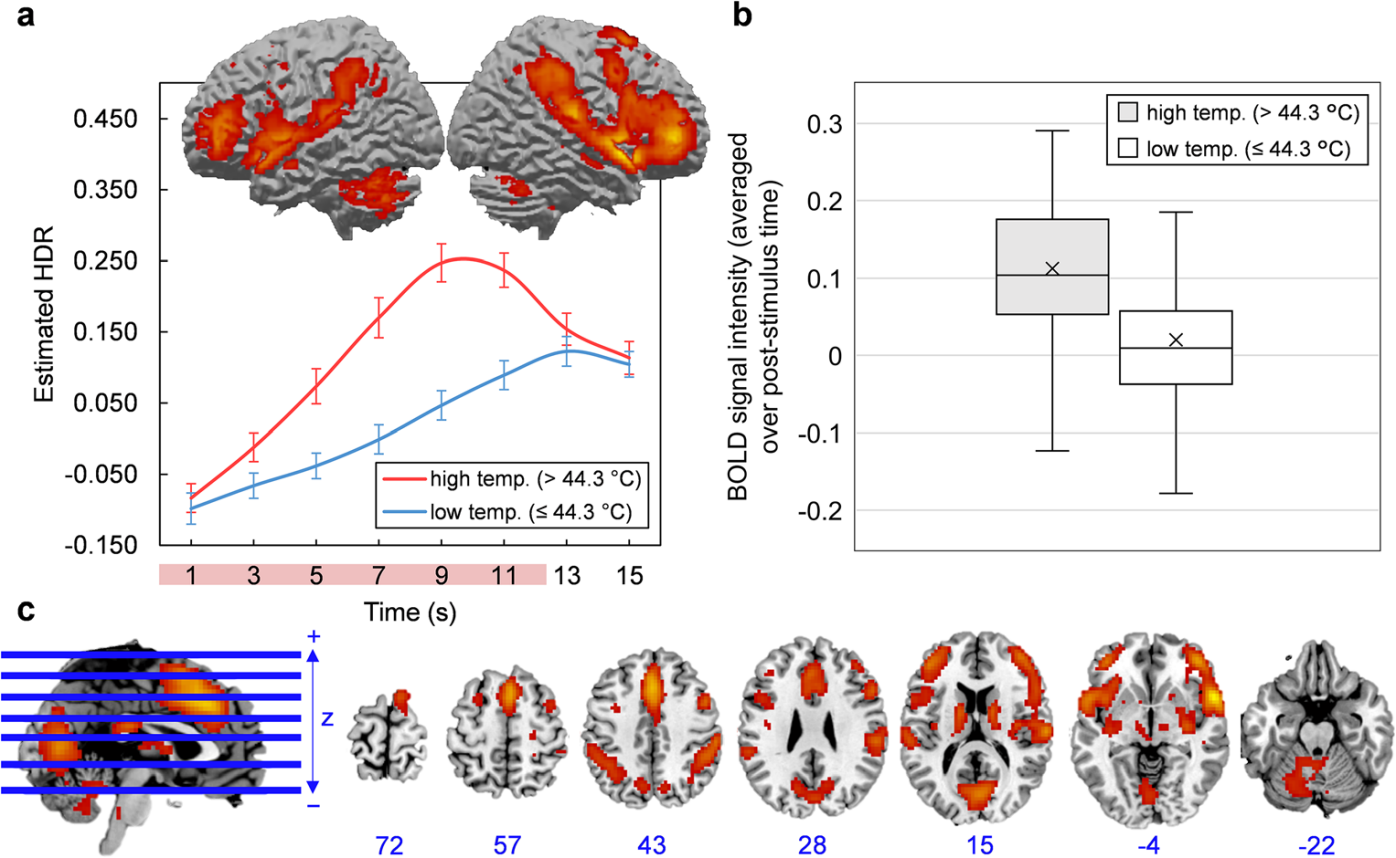
**a-c** Component 2. **a** Three-dimensional rendering of Component 2 (based on the top 10% of component loadings) and estimated HDRs associated with this network over the course of one thermal stimulation trial. The red bar placed over x-axis tick labels indicates the duration of a thermal stimulus (12.5 s). Estimated HDRs were obtained by averaging the FIR-based predictor weights for each condition level and plotting them as a function of post-stimulus time. Error bars given by standard error. HDR = hemodynamic response. **b** Boxplots illustrating the distributions of Component 2 BOLD signal across participants, for both high and low temperature stimuli. In both cases, BOLD signal was first averaged over the entire post-stimulus time interval for each participant and each condition. The mean is given by ×. **c** Horizontal cross-sections of Component 2 (only the top 10% of component loadings are shown). Positive loadings in red, threshold = 0.09, max = 0.21. No negative loadings. Blue values indicate the MNI coordinate of each slice in the z direction

**Fig. 4:**
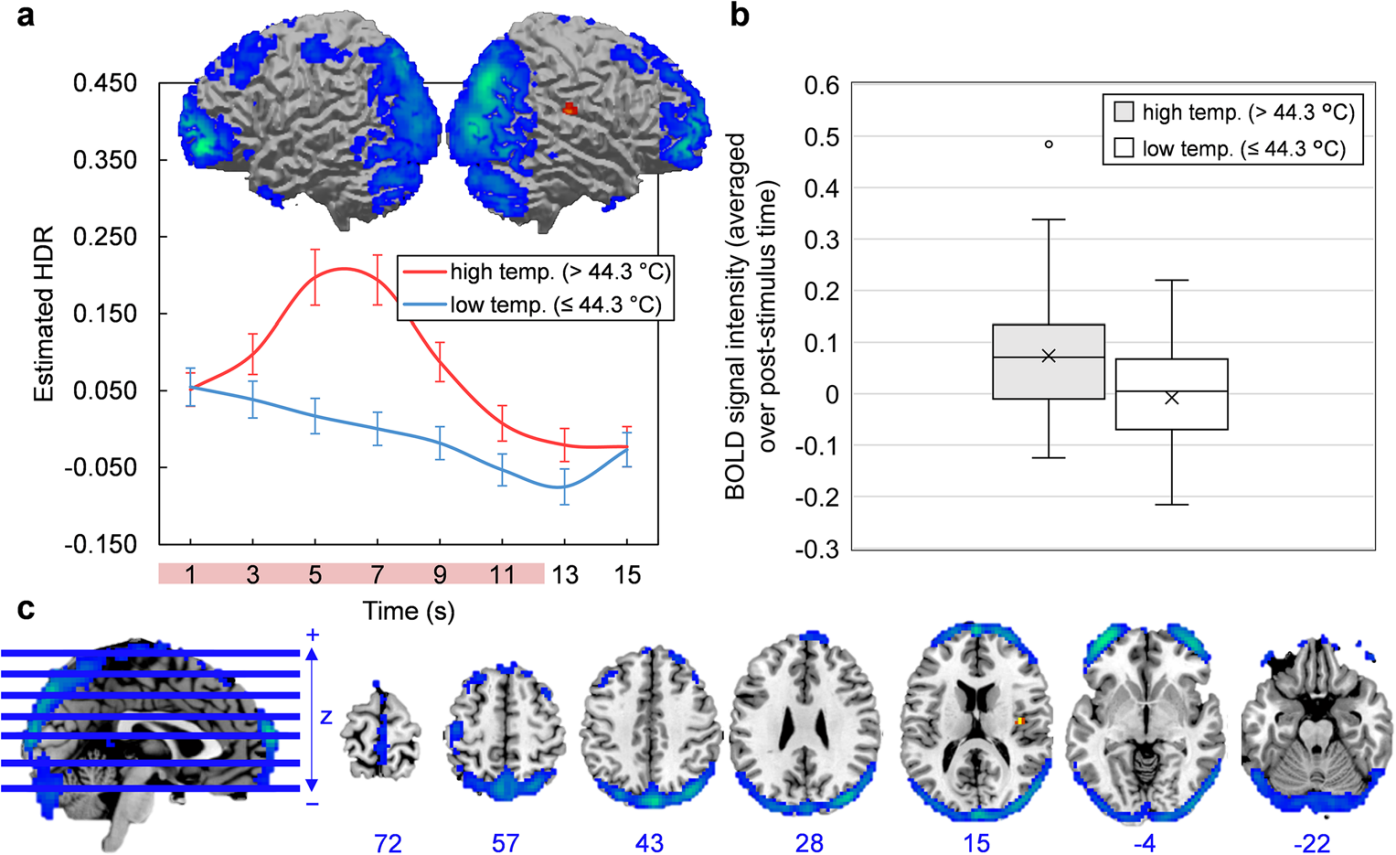
**a-c** Component 3. **a** Three-dimensional rendering of Component 3 (based on the top 10% of component loadings) and estimated HDRs associated with this network over the course of one thermal stimulation trial. The red bar placed over x-axis tick labels indicates the duration of a thermal stimulus (12.5 s). Estimated HDRs were obtained by averaging the FIR-based predictor weights for each condition level and plotting them as a function of post-stimulus time. Blue coloring indicates negative loadings; graphs should be interpreted as displaying the intensity of deactivation instead of activation. Error bars given by standard error. HDR = hemodynamic response. **b** Boxplots illustrating the distributions of Component 3 BOLD signal across participants, for both high and low temperature stimuli. In both cases, BOLD signal was first averaged over the entire post-stimulus time interval for each participant and each condition. The mean is given by ×. **c** Horizontal cross-sections of Component 3 (only the top 10% of component loadings are shown). Negative loadings in blue, threshold = −0.08, max = −0.16. Positive loadings in red, threshold 0.08, max = 0.09. Blue values indicate the MNI coordinate of each slice in the z direction

**Fig. 5:**
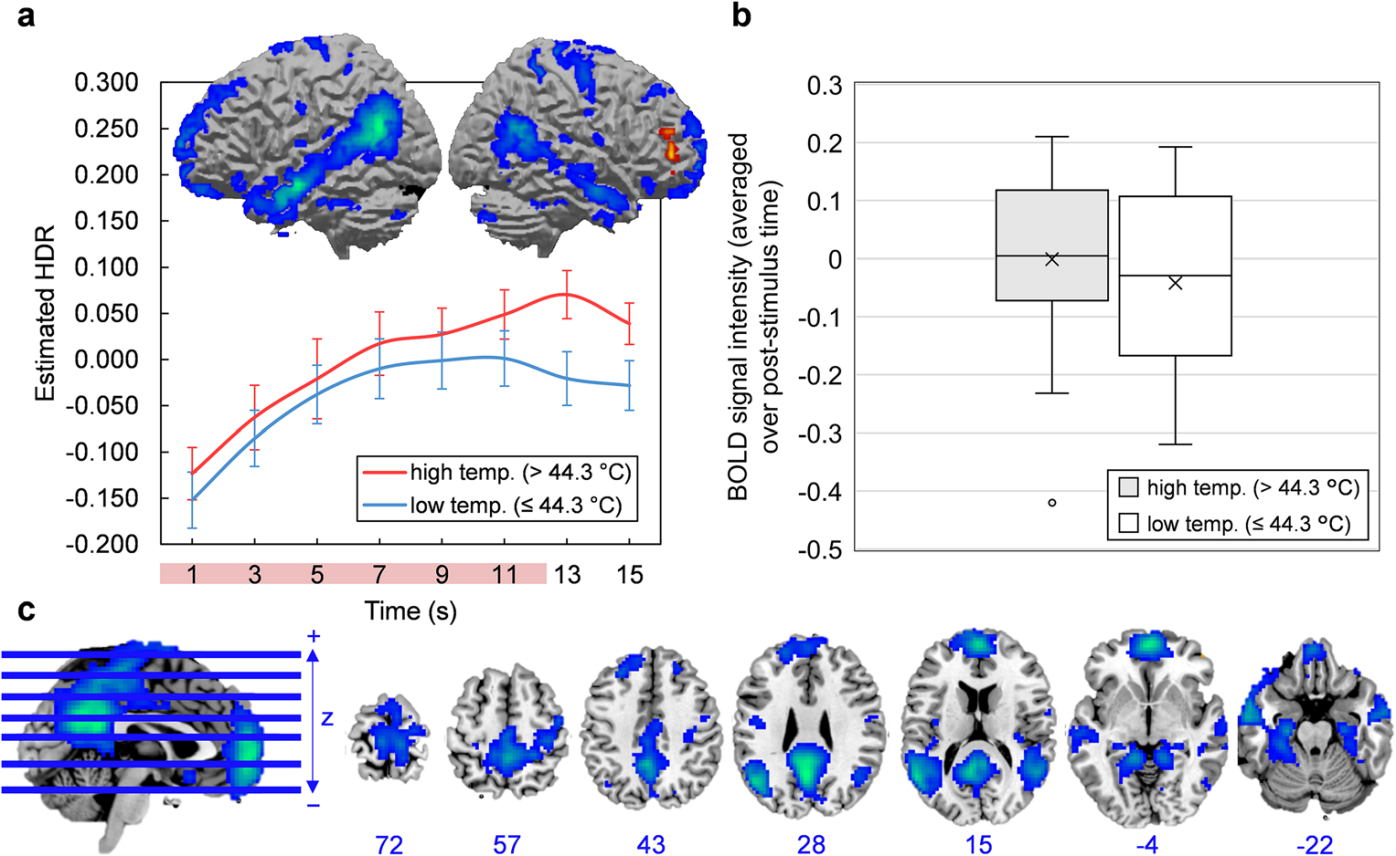
**a-c** Component 4. **a** Three-dimensional rendering of Component 4 (based on the top 10% of component loadings) and estimated HDRs associated with this network over the course of one thermal stimulation trial. The red bar placed over x-axis tick labels indicates the duration of a thermal stimulus (12.5 s). Estimated HDRs were obtained by averaging the FIR-based predictor weights for each condition level and plotting them as a function of post-stimulus time. Blue coloring indicates negative loadings; graphs should be interpreted as displaying the intensity of deactivation instead of activation. Error bars given by standard error. HDR = hemodynamic response. **b** Boxplots illustrating the distributions of Component 4 BOLD signal across participants, for both high and low temperature stimuli. In both cases, BOLD signal was first averaged over the entire post-stimulus time interval for each participant and each condition. The mean is given by ×. **c** Horizontal cross-sections of Component 4 (only the top 10% of component loadings are shown). Negative loadings in blue, threshold = −0.06, max = −0.14. Positive loadings in red, threshold 0.06, max = 0.07. Blue values indicate the MNI coordinate of each slice in the z direction

The scree plot of singular values indicated that four components should be extracted. Components 1, 2, 3, and 4 accounted for 21.47, 7.26, 4.95, and 3.74% of task-related variance in BOLD signal, respectively. Component images and estimated HDR shapes are displayed in Figs. 2, 3, 4, 5, along with network activation intensities for each temperature category (box plots).

Component 1 was primarily comprised of a) motor areas, including the primary motor cortex (M1), supplementary motor area (SMA) and cerebellum, b) visual areas, including the lateral occipital cortex (LO), and c) the primary somatosensory cortex (S1). Component 2 featured a frontoparietal activity pattern that included activation peaks in the anterior cingulate cortex (ACC), dorsolateral prefrontal cortex (dlPFC), anterior and posterior insula (aIns and pIns), and thalamus. Component 3 was limited to the outer edge of the cortex (specifically the frontal and occipital poles) and the longitudinal fissure and was mostly composed of negative loadings (i.e. it became deactivated during stimulation). This particular configuration was biologically untenable and resembled no established networks; it was most likely summarizing head movement that was coordinated with the application of thermal pain. Component 4 was characterized by deactivations in areas conventionally associated with the default mode network, including the posterior cingulate cortex (PCC), medial prefrontal cortex (mPFC), precuneuous and angular gyrus (AnG). Detailed anatomical descriptions for all networks are found in supplementary information (supplementary tables 1–3).

### Regressions

To relate these networks back to pain perception, we modelled variability in pain ratings as a linear function of activation intensities in all networks, both within and across individuals. The dataset used to model pain ratings consisted of pain ratings and network activation intensities for each temperature category (i.e. high and low) within each participant. Descriptive statistics (means and standard errors) for these data are found in supplementary table 4.

#### Within-Subject Pain

For within-subject pain, the model included temperature-dependent changes in network activation intensity for all functional networks identified by fMRI-CPCA (components 1, 2 and 4), and explained 39.7% of the variance in temperature-dependent changes in pain rating (*R*^2^ = 0.397; *F*(3, 27) = 5.9, *p* = .003), or 32.9% when adjusted for the number of predictors (${R}_{adjusted}^2$ = 0.329). This indicates that changes in pain ratings are predictable from changes in BOLD signal in the functional networks identified, according to:

$$ change\ in\ rating\sim - 138.94\left( C1\ BOLD\ signal\ intensity\ change\right)+ 201.39\left( C2\ BOLD\ signal\ intensity\ change\right)+ 112.07\left( C4\ BOLD\ signal\ intensity\ change\right)+ 40.78. $$

The accuracy of predicted scores was evaluated by taking the standard deviation of the residuals or the Root-mean-square error (RMSE), which was 18.27. As shown in Table 1, components 2 and 4 were the only statistically significant contributors (Component 2: *β* = 0.654, *p* = .001; Component 4: *β* = 0.482, *p* = .011). Also of note, Component 2 predicted increases in pain based on increases in its activation, whereas Component 4 predicted increases in pain based on increases in its *deactivation,* given that it consisted primarily of negative loadings (i.e. its intensity values represented deactivation, not activation, intensity).

**Table 1:** Within-subject pain: estimated regression coefficients and related statistics

*Predictor*	*b (S.E.)*	*β*	*t*	*p value*
(Intercept)	40.78 (7.19)		5.67	0.000
Component 1	-138.94 (79.41)	−0.29	−1.75	0.092
Component 2	201.39 (54.54)	0.65	3.69	0.001
Component 4	112.07 (40.77)	0.48	2.75	0.011

b = unstandardized regression coefficient, *β* = standardized regression coefficient, S.E. = standard error, *p*-values are two-tailed

**Fig. 6:**
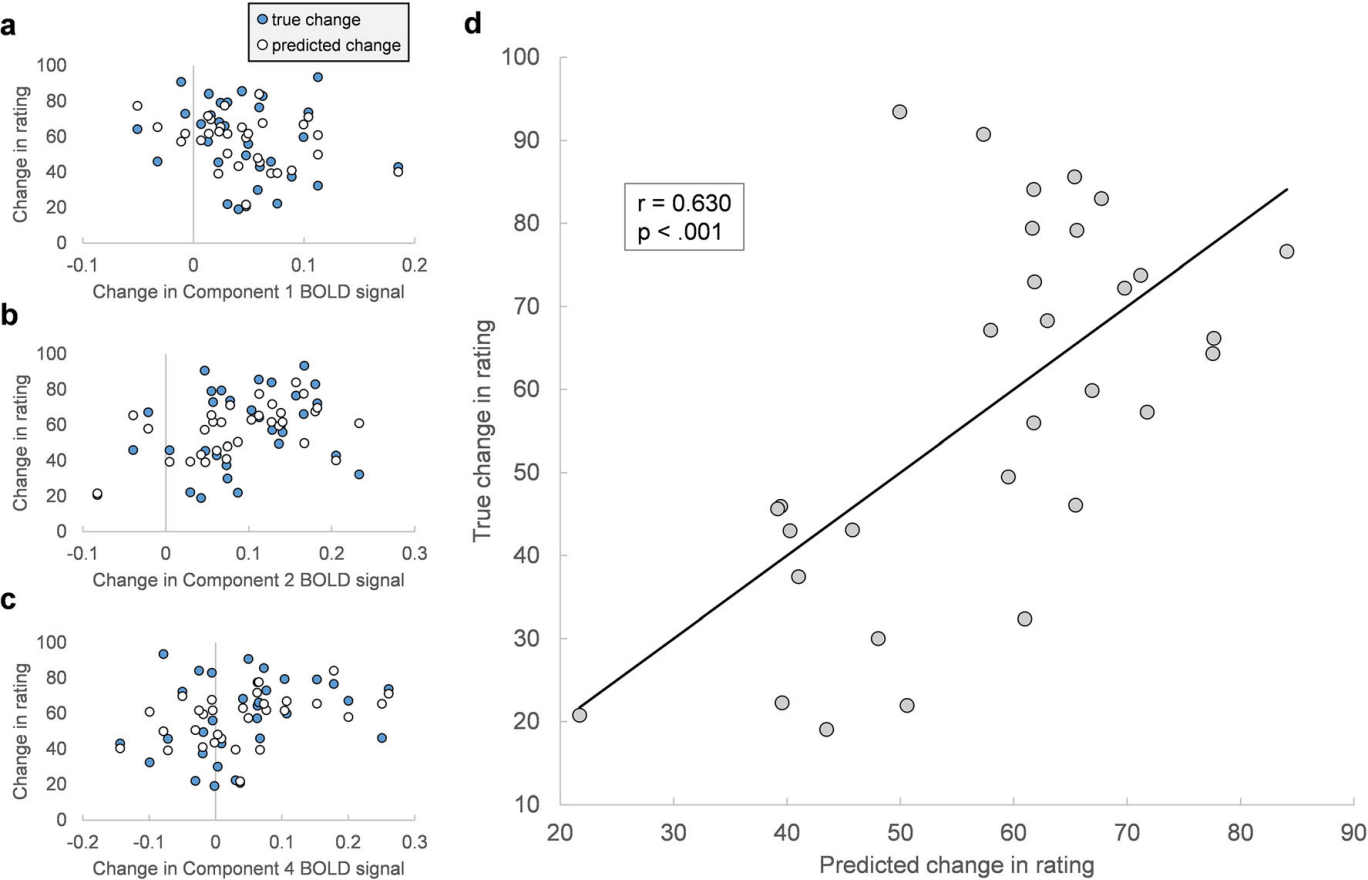
**a-d** Assessing model fit for within-subject pain. Temperature-dependent change in pain ratings plotted against change in BOLD signal for Component 1 (**a**), Component 2 (**b**), and Component 4 (**c**); true changes are shown alongside predictions made by the linear regression model. **d** True change in pain ratings plotted against change in ratings predicted by the model. The strength and significance of the relationship is given by Pearson r = 0.630, *p* < .001

To further evaluate model fit, changes in pain rating were plotted against changes predicted by the model in Fig. 6. The two variables were significantly correlated (*r* = 0.630, *p* < .001). Importantly, this model shows that the networks identified by fMRI-CPCA, as a whole, capture variations in pain perception at the *within-subject* level.

#### Between-Subject Pain

**Fig. 7:**
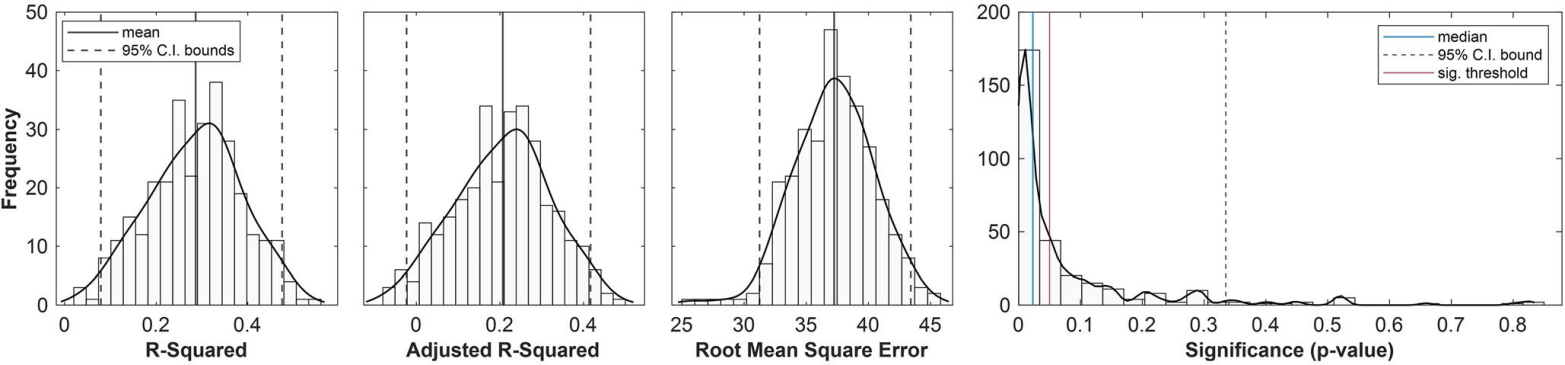
Assessing model fit for between-subject pain. Histograms, kernel density estimates, and average bootstrap estimates for R-squared, adjusted R-squared, Root-mean-square Error (RMSE) and model significance afforded by F-test (determines if a model fits significantly better than one based on a constant term only). Means and percentile intervals for the first three figures: $\overline{R^2}=$0.286, *CI*_*95%*_ = [0.079, 0.475]; $\overline{R_{adj.}^2}$= 0.207, *CI*_*95%*_ = [−0.023, 0.417]; $\overline{RMSE}$ = 37.238, *CI*_*95%*_ = [31.247, 43.404]. For model significance: 65.9% of models were significant at the .05 level, *p*_*median*_ = .023

Bootstrapped regression models of pain ratings, with all functional networks inputted as predictors, explained 28.6% of the variance in pain rating data on average ($\overline{R^2}$= 0.286, *CI*_*95%*_ = [0.079, 0.475]), or 20.7% when adjusted for the number of predictors (${R}_{adjusted}^2$ = 0.207). F-tests for the variance accounted for by each model revealed that 65.9% of the time, models were significant at the .05 level with a median *p* value of .023 (see Fig. 7).

**Fig. 8:**
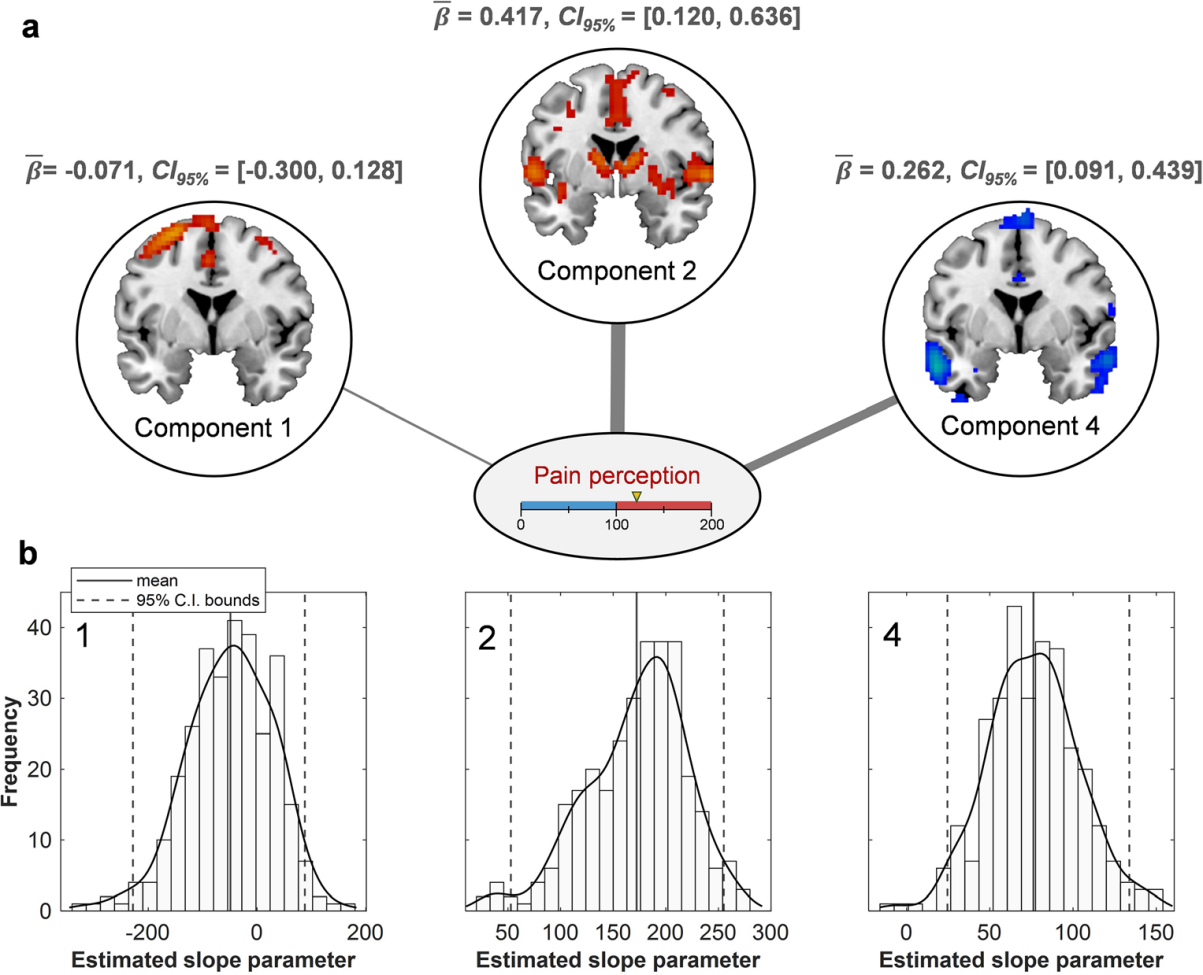
**a-b** Component contributions to pain. **a** Schematic depiction of each component’s contribution to pain ratings, according to bootstrap estimates of standardized regression coefficients (beta weights). Lines are proportional to the strength of their relation to pain. Component 4 is characterized by deactivation instead of activation; thus, its $\overline{\beta}$ value captures the strength of the relationship between Component 4 *deactivation* and pain perception. **b** Histograms, kernel density estimates, and average bootstrap estimates for unstandardized regression coefficients, with means and CI bounds. Component number indicated in the top left corner

The accuracy of predicted pain ratings was evaluated by averaging RMSE across all re-sampled models ($\overline{RMSE}$ = 37.24; Fig. 7). Estimated regression coefficients, their standard errors and confidence intervals are given in Table 2; components 2 and 4 were the only significant predictors of pain rating (Component 2: $\overline{\beta\ }$= 0.42, *CI*_*95%*_ = [0.12, 0.64]; Component 4: $\overline{\beta\ }$= 0.26, *CI*_*95%*_ = [0.09, 0.44]). Further, standardized coefficients showed that Component 2 made the most important contribution to the model, and its activation intensity was positively associated with pain ratings. By contrast, Component 4 *deactivation* intensity was positively associated with pain ratings. Figure 8 provides a schematic summary of these relationships between networks and pain perception. Overall, this model shows that—as a whole—the networks delineated by fMRI-CPCA are sensitive to *between-subject* variability in pain perception.

**Table 2:** Between-subject pain: estimated regression coefficients and related statistics

	Unstandardized coefficient		Standardized coefficient (β)	
*Predictor*	*Average Bootstrap Estimate (S.E.)*	*Percentile CI (95%)*	*Average Bootstrap Estimate (S.E.)*	*Percentile CI (95%)*
(Intercept)	73.67 (16.81)	[36.14, 103.13]		
Component 1	−48.95 (78.65)	[−228.15, 87.88]	−0.07 (0.11)	[−0.30, 0.13]
Component 2	172.26 (47.66)	[52.76, 255.13]	0.42 (0.12)	[0.12, 0.64]
Component 4	76.24 (27.04)	[24.54, 133.80]	0.26 (0.09)	[0.09, 0.44]

S.E. = standard error, i.e. the standard deviation of the corresponding bootstrap distribution

**Fig. 9:**
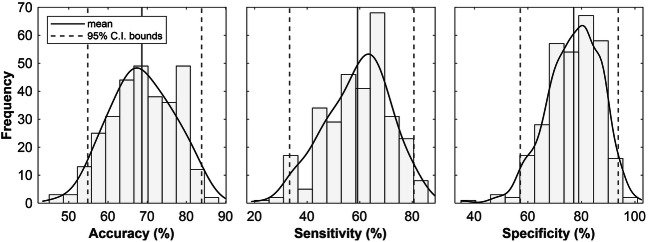
Classification performance. Histograms, kernel density estimates, and average bootstrap estimates of model accuracy (Mean_acc._ = 68.83, *CI*_*95%*_ = [54.84, 83.87]), sensitivity (Mean_sens._ = 59.17, *CI*_*95%*_ = [33.33, 80.63]) and specificity (Mean_spec._ = 77.10, *CI*_*95%*_ = [57.14, 93.75])

To evaluate the model’s ability to differentiate between painful and non-painful states, we converted true ratings and predicted ratings into binary categories (i.e. pain or non-pain, as described in method) and calculated accuracy, sensitivity and specificity of classification for every resampled model; Supplementary Figure 1 demonstrates this procedure for one bootstrap sample. Empirical bootstrap distributions for accuracy, sensitivity and specificity are shown in Fig. 9. Estimates (obtained by averaging) were 68.83%, 59.17% and 77.10%, respectively. Confidence intervals show that only accuracy and specificity were significantly higher than chance level (50%). This indicates that the overall accuracy of the model is driven by its specificity, or its ability to correctly identify non-pain (more precisely, specificity is the fraction of pain ratings below 100 correctly classified as “non-pain”). It appears that the predicted ratings tend to better approximate ratings below the pain threshold than ratings above it, such that when ratings are converted to the binary variable “pain” or “non-pain”, ratings above the threshold are more often miscategorized than ratings below it.

## Discussion

In this study, distinct functional connectivity networks for pain were revealed by fMRI-CPCA. The networks encompass a variety of brain regions consistently active in response to pain, including MI, SMA, cerebellum and SI (Component 1), the ACC, insular cortex, and thalamus (Component 2), and mPFC, hippocampus, para-hippocampus and precuneus (Component 4) (Apkarian et al., 2005; Atlas et al., 2014; Schweinhardt & Bushnell, 2010). Within participants, changes in perceived intensity related to low and high temperatures were associated with the magnitude of change in BOLD across networks. While falling short of accurate classification, the magnitude of BOLD activation in functional networks was significantly associated with pain intensity between participants. Future development of fMRI-CPCA in the context of pain is warranted to further explore the brain in pain.

Over and above capturing the activation of known pain regions, fMRI-CPCA integrated these brain regions into multiple functional networks. Although the specific parcellation observed here is unique, it is largely congruent with current perspectives on pain-related networks. In particular, evidence from PET and fMRI suggests that pain-activated regions are segregated into at least four distinct sub-networks: a sensory network for stimulus localization and intensity coding (Davis & Moayedi, 2013; Hofbauer et al., 2001; Peyron et al., 1999), an affective network for generating the aversive, unpleasant quality of a stimulus (Davis & Moayedi, 2013; Peyron et al., 2000; Wilcox et al., 2015), a cognitive network for attending to, anticipating and remembering the stimulus (Davis & Moayedi, 2013; Peyron et al., 1999; Wilcox et al., 2015), and a network of motor regions for pain avoidance (Davis & Moayedi, 2013; Wilcox et al., 2015).

### Component 1 (Sensorimotor Response)

Component 1, with prominent activation peaks in MI, SMA, and cerebellum, is most aptly described as a sensory and motor network. In the context of thermal stimulation, sensation and motor output may be related to an instinctive flexing or bracing, or a desire to move, in response to intense stimuli (Davis & Moayedi, 2013; Davis et al., 2002). Hemodynamic response shapes (HDRs) for Component 1 showed that activation was in fact exclusive to higher intensity stimuli (temperatures above 44.3 °C). Component 1 also included prominent activations in SI, which, as a key cortical aspect of the lateral nociceptive system, is one of the first recipients of ascending pain signals through the spinothalamic tract (Davis & Moayedi, 2013; Fomberstein et al., 2013; Vierck et al., 2013; Yam et al., 2018). SI’s inclusion in Component 1 suggests that, during thermal stimulation, motor processes are prioritized and closely coordinated with sensory-discriminative functions (e.g. determination of stimulus location and intensity). In theory, such close communication would be necessary for an effective pain avoidance response when stimulus intensity reaches noxious levels (Postorino et al., 2017). This plausible role of Component 1 in generating pain-induced motor commands remains to be further explored; follow-up studies would benefit from monitoring physical movements in conjunction with other variables, allowing for the precise relationships between Component 1 activation intensity, stimulus intensity (e.g. temperature), motion, and pain perception to be determined.

A novel observation from fMRI-CPCA is the temporal overlap between visual areas and sensory-motor coupling, evidenced in Component 1. Their detection is likely an idiosyncratic capture of fMRI-CPCA, which avoids using regions-of-interest to spatially constrain the analysis. In fact, the anatomy of Component 1 replicates previous applications of fMRI-CPCA in other domains—specifically, it resembles a network consistently associated with sensorimotor response processes, featuring activations in lateralized MI, SMA, SI, cerebellum, and visual areas including the lateral occipital cortex (LO; Goghari et al., 2017; Larivière et al., 2017; Metzak et al., 2011). In this case, sensorimotor-visual coupling was likely observed because of screen-related cues that coincided with stimulus presentation.

### Component 2 (Attentional Pain Network)

In agreement with previous studies, Component 2 incorporated a large number of regions involved in pain, and combined sensory, affective and cognitive sub-networks (Davis & Moayedi, 2013; Wilcox et al., 2015). For example, the most prominent activation peaks were found in SII and posterior insula (pIns; sensory-discriminative regions), dACC, aIns, and thalamus (affective-motivational regions), and dlPFC and IPL (cognitive-evaluative regions; Peyron et al., 1999; Wilcox et al., 2015). Based on this, Component 2 could reflect a unification of sensory, affective and cognitive processes (Melzack & Casey, 1968) into a coordinated pain response.

The blending of sub-networks is likely facilitated by their inter-connectivity at rest, provided by common nodes in ACC and aIns that serve as relay sites between sub-networks (Wilcox et al., 2015). Importantly, the ACC and aIns are engaged in non-specific “salience detection”, where stimuli are selected based on their behavioural relevance, and attentional systems are primed to enable an effective response (Legrain et al., 2011; Menon, 2015). Such a “salience network” receives axonal projections from sensory areas like the pIns, which are thought to provide the aIns with incoming sensory information (Menon & Uddin, 2010). The pattern of activation observed in Component 2 captures both attentional systems (i.e. the cognitive sub-network of pain) and sensory-discriminative elements like SII and pIns. Thus, Component 2 may represent a salience network-mediated response to salience—in this case thermal stimulation—or more precisely a sequential activation of sub-networks, i.e. sensory systems activate the salience network, which then activates cognitive systems for sustained attention. The directionality of sub-network relations is a matter of speculation, but it presents an interesting question for future investigation. Additionally, the putative attentional function of Component 2 may be further explored by analyzing its pain-induced response during experimental manipulations of attentional demand or stimulus salience; a larger effect of attention on network response than stimulus temperature would suggest an attentional role.

### Component 4 (Default-Mode Network)

The tendency for brain areas to become deactivated during a task and engaged at rest gave rise to the original concept of the “default mode of brain function” (Shulman et al., 1997; Raichle et al., 2001). Since being originally characterized, research has emerged documenting the overall functional contributions of the default-mode network (DMN) to human behavior, including its relevance to mind-wandering, self-referential thought, mentalizing and semantic processing (Andrews-Hanna, 2012; Andrews-Hanna et al., 2014; Christoff et al., 2009).

Component 4 was comprised of deactivations in regions conventionally associated with the DMN, including the PCC, the AnG, and the amPFC. Such a deactivation departs from the proposed sub-network scheme discussed above (i.e. sensory, affective, cognitive, and motor sub-networks of pain; Davis & Moayedi, 2013; Wilcox et al., 2015). However, the DMN has also been implicated in pain and so its detection here is not entirely unexpected. In chronic pain disorders, for example, the DMN shows a number of anatomical-functional alterations, including fragmentation between frontal and posterior regions (Baliki et al., 2014), and strengthening of functional connections to aIns (Baliki et al., 2014; Loggia et al., 2013). In healthy individuals, heat-induced *deactivations* in several DMN regions have been reported (Kong et al., 2010), while some regions, like the hippocampus and precuneus, also predict pain ratings (in addition to stimulus intensity) by the magnitude of their deactivation (Atlas et al., 2014).

As others have argued, pain-induced deactivations in the DMN may be part of an attentional response to pain (Kucyi et al., 2013; Kucyi & Davis, 2015), where the DMN suppresses as attentional networks (e.g. Component 2) engage. This type of antagonistic relationship between the DMN and attentional networks has been documented extensively outside of pain imaging, along with the DMN’s “task-negative” tendencies (Anticevic et al., 2012; Peng et al., 2018). Future research would benefit from an analysis of DMN response to pain in the context of attentional manipulations. Alternatively, attention levels during a stimulus could be monitored to allow for an analysis of the relationships between DMN deactivation, DMN-Component 2 antagonism, pain perception and attention.

Also of note, several DMN regions, including the mPFC, hippocampus, and precuneus, have been associated with the regulation of pain (Goffaux et al., 2014; Schweinhardt & Bushnell, 2010). Their involvement implies a potential role of the DMN, which might accomplish regulation by interacting with the periaqueductal gray (PAG)—part of a descending pathway for pain control—through the mPFC (Kucyi et al., 2013). Thus, chronic pain disorders may be related, in part, to deficits in pain regulation caused by alterations to the DMN. This possibility requires further investigation and presents an important research objective due to its implications for chronic pain treatment.

### Estimating Pain Within and Between Participants

Among intended applications of neuroimaging in the field of pain is the development of models to accurately classify an individual in pain. Previous attempts of this nature have adopted multivariate pattern analysis (MVPA; Haynes, 2015; van der Miesen et al., 2019). In brief, MVPA uses machine learning algorithms to model behavioural responses (either ordinal or continuous variables) as a function of multiple voxels (or “features”) considered simultaneously (Moayedi et al., 2018; van der Miesen et al., 2019); predictions or classifications of mental states are then generated on independent “testing” data based on model parameters learned in the “training” set (Rosa & Seymour, 2014). In one notable study applying MVPA, a network of regression weights distributed over pain regions (the “neurologic pain signature” or NPS) tracked physical pain intensity *between* individuals (Wager et al., 2013; Woo et al., 2015). Perhaps even more remarkable is that physical pain could be accurately distinguished from other types of pain (e.g., social; Wager et al., 2013).

In this study, regression models provided some insight into the capacity of networks detected through fMRI-CPCA to be used for pain prediction, as components 1, 2 and 4 were significantly associated with pain ratings both within and between participants. Importantly, this was not a predictive model (networks were used to model in-sample ratings with no predictions generated on new or held-out data), and the findings should not be interpreted as direct evidence of prediction ability. However, networks did show potential to be used in predictive analyses given that in-sample estimation was moderately accurate, and, importantly, results were achieved without any a priori selection of brain regions, reflecting a distinct advantage of fMRI-CPCA compared to other approaches.

Of all networks, Component 2 was most strongly related to pain perception; the relationship was positive and consistently accounted for the largest proportion of within- and between-subject variability in pain. The value of Component 2 for predicting pain is intuitive, insofar as brain regions included in this network represent sensory, affective, and cognitive dimensions of pain (Melzack & Casey, 1968). The DMN was also important for pain estimation, with the magnitude of its deactivations being significantly related to perceived pain intensity, both within and between participants. The relationships of both networks to pain are corroborated by previous work that has identified several Component 2 regions—including SII, aIns, dACC, left cerebellum, and IPL—and DMN regions—including hippocampus and precuneus—as explicit mediators of pain (i.e. they mediate the relationship between stimulus intensity and pain rating; Atlas et al., 2014).

The intensity of activation in Component 1 was unrelated to the intensity of perceived pain, mirroring the behaviour of SI itself, which codes pain information primarily in terms of sensory-discriminative attributes (Moulton et al., 2012). This aspect of Component 1 (i.e. its independence from pain perception) is corroborated by mediation analyses that demonstrate a preference of sensory cortex and cerebellum to stimulus intensity over pain report (Atlas et al., 2014), and implies that motor systems are mobilized in accordance with stimulus properties only; the perception of pain occurs elsewhere, and the intensity of motor commands is, on its own, an unreliable proxy for the intensity of that perception.

Despite significant associations, when converted into a classifier the model discriminated between pain and warmth with an accuracy of only 68.83%. While significantly greater than chance, sensitivity and specificity were low (estimated at 59.17% and 79.10%, respectively). Still, comparisons between components 1, 2 and 4 and the existing “neurological pain signature” (NPS) reveal a high degree of overlap. Common regions include aIns, pIns, supramarginal gyrus, thalamus, and IPL. Further, the NPS included negative predictive weights in regions that were deactivated in Component 4, including PCC, precuneus and mPFC (Wager et al., 2013). These anatomical similarities raise the possibility that accurate predictions of pain could be generated from components 1, 2 and 4 if specific regional activations (compared to an overall estimate of activation in the entire network) were accounted for using MVPA (Allefeld & Haynes, 2015). By avoiding spatial averaging, MVPA accounts for signal non-uniformities between voxels, and exploits these differences in response signal as a source of predictive information (Hebart & Baker, 2018).

Crucially, the predictive potential shown by components indicates that fMRI-CPCA may provide a useful tool for determining appropriate anatomical targets for MVPA. This is important because a critical step in the MVPA framework is the selection of “features” with which to train the machine learning algorithm (Rosa & Seymour, 2014). Features are typically a subset of voxels, whose activations will be related to the behavioural response by the algorithm (Allefeld & Haynes, 2015), and are selected from a region- or regions-of-interest (based on prior knowledge) or from the entire brain using dimensionality reduction techniques like PCA (van der Miesen et al., 2019). Restricting the analysis to relevant regions is important to mitigate the problem of features exceeding the number of observations, which may lead to overfitted models and interpretive challenges (van der Miesen et al., 2019). In the case of the NPS, features were selected a priori from a collection of well-established pain regions (Wager et al., 2013). By contrast, fMRI-CPCA would allow features to be selected from the predominant functional networks involved in pain perception, without relying on prior assumptions about relevant spatial or temporal response patterns. fMRI-CPCA thus provides an opportunity to select connectivity-based features (Rosa & Seymour, 2014) that are unbiased, data-driven and task-related.

As a final point, results from multiple regressions are not only relevant to pain prediction, but also reflect on network functions proposed earlier, specifically the roles of Component 2 and the DMN as attention networks. In the regressions, Component 2 and the DMN displayed opposite relationships to pain; higher pain was associated with greater activation in Component 2 but greater suppression in the DMN, both within and between participants. This is an extension of the pattern shown by estimated HDRs, where Component 2 became active during stimulation while the DMN became suppressed. Together, these findings suggest that Component 2 and the DMN assume an antagonistic configuration during pain, and that greater antagonism (i.e. greater separation in terms of activation) equates to a heightened perception of pain.

Based on neuroimaging literature, this antagonism is likely indicative of an ongoing attentional response. Component 2 included known salience network hubs in ACC and aIns, as well as cognitive pain regions associated with attention to pain, and the DMN’s role in attention has been well-documented. For example, the DMN tends to form anticorrelated relationships with frontoparietal attention networks during cognitively demanding tasks (Dixon et al., 2017; Menon, 2015; Sridharan et al., 2008), with greater deactivation predicting improved task performance (Anticevic et al., 2012). Furthermore, attention deficits are generally associated with increased DMN activation (Bonnelle et al., 2011; Weissman et al., 2006; Danckert & Merrifield, 2018). In the context of pain, DMN deactivation is especially pronounced when participants report *attending* to pain, and less so when participants mind-wander away from pain (Kucyi et al., 2013). Thus, the deactivation of DMN observed here likely signifies attention to pain. The simultaneous activation of Component 2—which included several regions known to be involved in attention—mirrors the stereo-typical antagonism between DMN and frontoparietal networks that underlies attention (Anticevic et al., 2012). In sum, these networks appear to contribute to pain perception by working together, in an anticorrelated fashion, as part of an attentional response process; the greater the attention, the greater the antagonism between networks and the greater the pain intensity.

### Technical Considerations

Based on the literature discussed in sections above, it is possible to infer the functionality of each network. However, these inferences are speculative and are not necessarily validated by any direct experimental evidence obtained here; instead they rely on prior notions about the functional contributions of regions or networks detected. Importantly, the fMRI-CPCA framework provides an opportunity to more robustly characterize network function during a task. This is done by comparing the HDRs estimated for each network across task conditions to determine how different combinations of independent variables impact network behaviour. Statistical comparisons can be made using repeated-measures ANOVAs, with within-subject factors given by time and independent variables of interest (e.g. temperature level in this study). By carefully manipulating experimental conditions, cognitive processes can be dissociated from each other, and by interpreting main and interaction effects of factors on HDRs, networks can be related to *specific* aspects of cognition operationalized by task conditions. Comparisons can also be made between populations of interest by adding between-subject factors that define group membership. In this way, network alterations or deficits associated with diagnostic categories—such as chronic pain disorders—can be investigated.

It should be noted that the HDRs estimated by fMRI-CPCA are well-suited to making inferences about cognitive function; this is because fMRI-CPCA uses Finite Impulse Response (FIR)-basis sets to encode brain activity associated with task-timing, which are essentially dummy regressors for stimulus presentation timing that make no assumptions about the shape of the expected response. For this reason, the technique detects responses (and by extension, functional networks) elicited by cognitive processes that may go unnoticed in more traditional analyses, where the expected response is produced by convolving stimulus functions with canonical hemodynamic response functions (Henson & Friston, 2007; Henson et al., 2001; Lindquist, 2008; Lindquist et al., 2009). Detailed analysis of HDR shapes evoked in components 1, 2 and 4, under different experimental conditions, is therefore warranted to achieve a robust determination of network function.

### Limitations

Our study has a number of limitations to consider. First, we did not include a protocol for model validation when evaluating pain predictions and classifications made with multiple linear regression; the ability of the model to predict or classify pain in independent samples therefore remains unverified. Validation techniques—including cross-validation, hold-out validation, or bootstrapping—are common practice in decoding analyses to ensure that models generalize to out-of-sample data (Kohavi, 1995; van der Miesen et al., 2019). We did not apply these here because of properties of the data (primarily its small sample size of 30), which made a conventional approach challenging (e.g. some subjects never reported a stimulus as painful). Future research is needed to formally validate the pain predictive value of these networks.

In theory, the regression model obtained here is generalizable to new and independent data. The basic procedure would involve application of the regression coefficients obtained to the network activations estimated in a new individual to generate a predicted pain rating. This would require first obtaining an individual’s activation data during a thermal pain task, analysing their brain activity using fMRI-CPCA, and “classifying” the networks elucidated through in-house programs recently developed to determine which of the new individual’s networks most closely match with the networks that inform the current model (Percival et al., 2020). The HDR shapes associated with the correct networks would have to be averaged across an appropriate time interval (or an equivalent time interval to the current study) to generate estimates of network activation intensity, and the regression model obtained here would then be applied to network activation intensities to generate a predicted pain rating. The classification procedure referred to above has been utilized previously and involves correlating the loadings of networks obtained with the loadings of “template” images of networks, across a set of characteristic slices that define the individuality of a network. Each network identified in a new individual would have to be correlated with the template images of networks obtained in this study to determine the strongest matches. Ultimately, this classification procedure would aid in selecting networks whose activations (averaged over post-stimulus time) would then be subjected to the regression model in order to generate a pain prediction.

To address this first limitation (lack of model validation), future research may use larger datasets to re-conduct the current study with the addition of a validation protocol, or test the current model in new and independent data according to the procedure outlined above. That said, predictions based on these networks are likely to be improved if signal differences within sub-networks and regions of components are accounted for by using pattern-based analyses like MVPA, instead of constructing models based on a whole-network index of activation (i.e. estimated HDRs for entire networks).

A second limitation is that we included stimuli not rated as painful (based on the 100-point pain threshold specified by the VAS scale) in both network extraction via fMRI-CPCA and regression models of pain ratings. For network extraction, this means that networks delineated were composed of voxels that remained functionally-connected across non-painful *and* painful stimulation; in this way, any voxels that became incorporated into the networks—or any new networks that were formed—during painful stimuli only were potentially missed by the analysis. For regression models, it raises the possibility that networks were related to warmth more so than pain perception. This would be the case if model-based predictions of ratings below the pain threshold (i.e. warmth) were consistently better than those of ratings above the pain threshold (i.e. pain).

### Conclusion

Overall, this study has contributed to neuroimaging research on pain by elucidating three functional networks evoked by thermal stimulation: a sensorimotor response network for immediate pain avoidance (Component 1), a frontoparietal attention network mobilized by salience detection processes (Component 2), and the default-mode network (Component 4). Of these, attention and default-mode networks were related to pain perception both within and between participants. From a purely technical perspective, this study validates fMRI-CPCA within the domain of pain research for the first time, highlighting advantages compared to existing approaches, including that the parcellation of multiple task-related networks is accomplished without a priori selection of regions-of-interest (i.e. no assumptions about spatial properties of networks). Moreover, fMRI-CPCA does not rely on models that assume specific HDR shapes to identify task-related activity; instead, HDRs are predicted using FIR basis functions, which simply specify an interval during which task-relevant activity is expected to occur. In this way, fMRI-CPCA detects HDRs (and potentially networks) elicited by cognitive processes that may be unaccounted for in conventional analyses.

More generally, the findings obtained provide a foundation from which to further investigate these networks, their proposed functions and their pain predictive value. The networks identified (especially the attention and default-mode networks) may have implications for pain treatments, if they can be targeted successfully with strategies based on neuromodulation (Alo & Holsheimer, 2002), behavioural therapy (Eccleston et al., 2013), or real-time fMRI feedback (Chapin et al., 2012), for example. Further, validated pain predictions can be generated from these networks and potentially refined by applying MVPA within network boundaries. Patterns delineated through MVPA may ultimately serve as objective measures of pain, which are of crucial importance to effective pain management in patients unable to self-report their pain.

## Information Sharing Statement

The thermal stimulation fMRI data that support the findings of this study were originally published by Woo et al. (2015) and are available from openneuro.org, https://openneuro.org/datasets/ds000140/versions/00001. Accession number ds000140.

The fMRI-CPCA program is available from https://www.nitrc.org/projects/fmricpca; codes are implemented in MATLAB using a graphical user interface. Custom code for within- and between-subject regression models can be accessed from GitHub, https://github.com/MatteoDamascelli/Multiple-Functional-Brain-Networks-Related-to-Pain-Perception-Revealed-by-fMRI.

## Supplementary Information

Supplementary Fig 1 **a-c** Illustration of the process by which classification metrics were derived for each resampled linear model, using a single (demonstrative) bootstrap sample. (a) True pain ratings are plotted against pain ratings predicted by the model that was computed on the sample; dotted lines separate painful from non-painful cases, according to the 100-point threshold specified by the VAS scale, and green areas define cases that were correctly classified into either category (pain or no pain). (b) Scaled down version of the scatterplot in (a), where the value in each quadrant represents the percentage of total cases that were contained within it; again, green areas define cases that were correctly classified. Accuracy is the sum of all correct classifications—in this case 77.4. (c) Scaled down version of the scatterplot in (a), but this time the top two quadrants contain the percentage of pain cases that were either correctly classified as pain (top right) or incorrectly classified as non-pain (top left), and the bottom two quadrants contain the percentage of non-pain cases that were either correctly classified as non-pain (bottom left) or incorrectly classified as pain (bottom right). Thus, the top right provides a measure of sensitivity, i.e. how effective the model is at recognizing pain, and the bottom left provides a measure of specificity, i.e. how effective the model is at recognizing non-pain. All three metrics (accuracy, sensitivity, specificity) were generated for every sample, and were then estimated from their empirical bootstrap distributions (PNG 913 kb)

High resolution image (TIF 1227 kb)

ESM 2 (PDF 73 kb)

ESM 3 (PDF 99 kb)

ESM 4 (PDF 108 kb)

ESM 5 (PDF 30 kb)

ESM 6 (DOCX 25.6 kb)

## Data Availability

Data are available on a public repository online, and are freely accessible. A link can be found in the Information Sharing Statement.
