# Supplementary figures and images for: Multiple Functional Brain Networks Related to Pain Perception Revealed by fMRI
Source: Neuroinformatics. 2021 Jun 8;20(1):155–72. doi: 10.1007/s12021-021-09527-6 (PMC9537130; doi:10.1007/s12021-021-09527-6)

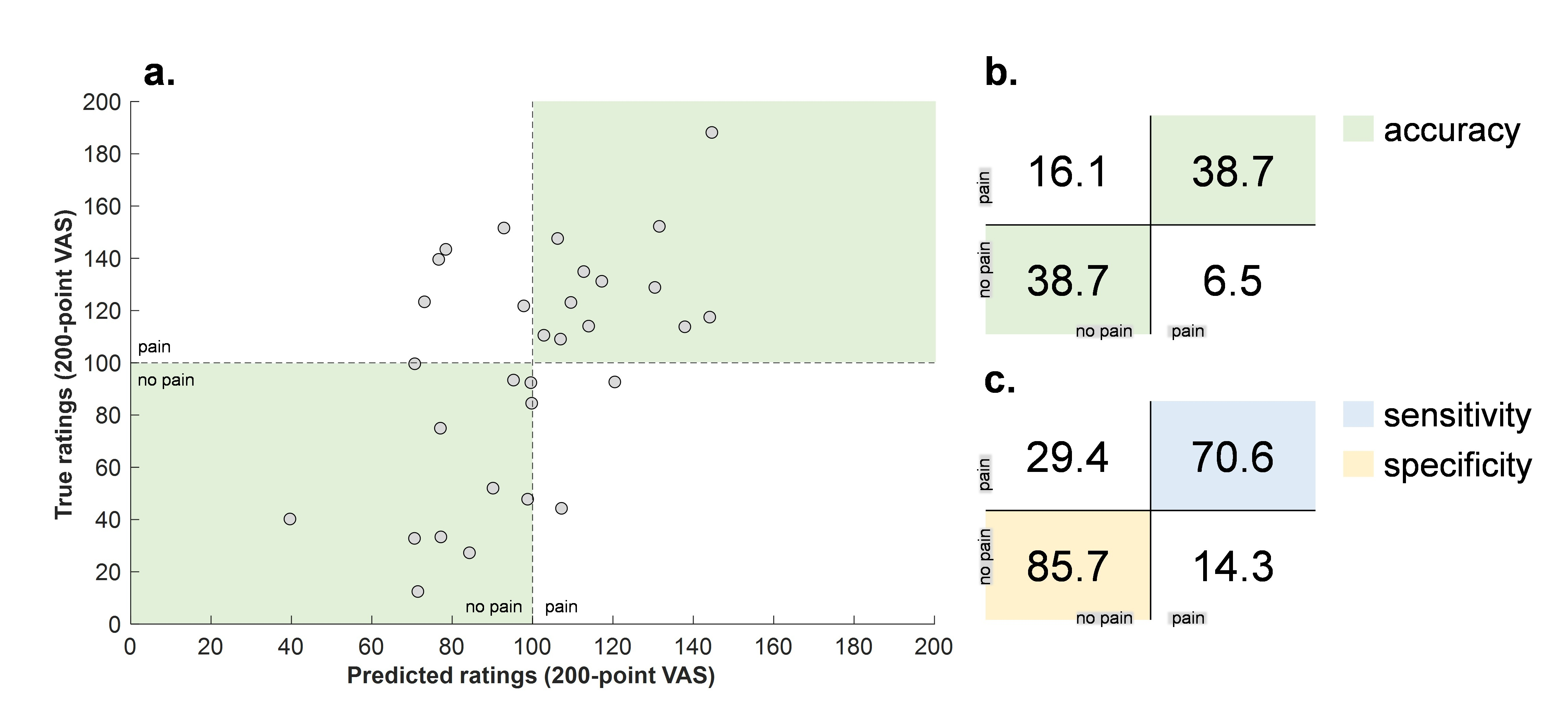

Supplement: Supplementary file 1 — a-c Illustration of the process by which classification metrics were derived for each resampled linear model, using a single (demonstrative) bootstrap sample. (a) True pain ratings are plotted against pain ratings predicted by the model that was computed on the sample; dotted lines separate painful from non-painful cases, according to the 100-point threshold specified by the VAS scale, and green areas define cases that were correctly classified into either category (pain or no pain). (b) Scaled down version of the scatterplot in (a), where the value in each quadrant represents the percentage of total cases that were contained within it; again, green areas define cases that were correctly classified. Accuracy is the sum of all correct classifications—in this case 77.4. (c) Scaled down version of the scatterplot in (a), but this time the top two quadrants contain the percentage of pain cases that were either correctly classified as pain (top right) or incorrectly classified as non-pain (top left), and the bottom two quadrants contain the percentage of non-pain cases that were either correctly classified as non-pain (bottom left) or incorrectly classified as pain (bottom right). Thus, the top right provides a measure of sensitivity, i.e. how effective the model is at recognizing pain, and the bottom left provides a measure of specificity, i.e. how effective the model is at recognizing non-pain. All three metrics (accuracy, sensitivity, specificity) were generated for every sample, and were then estimated from their empirical bootstrap distributions (PNG 913 kb) [file 12021_2021_9527_Fig10_ESM.png]

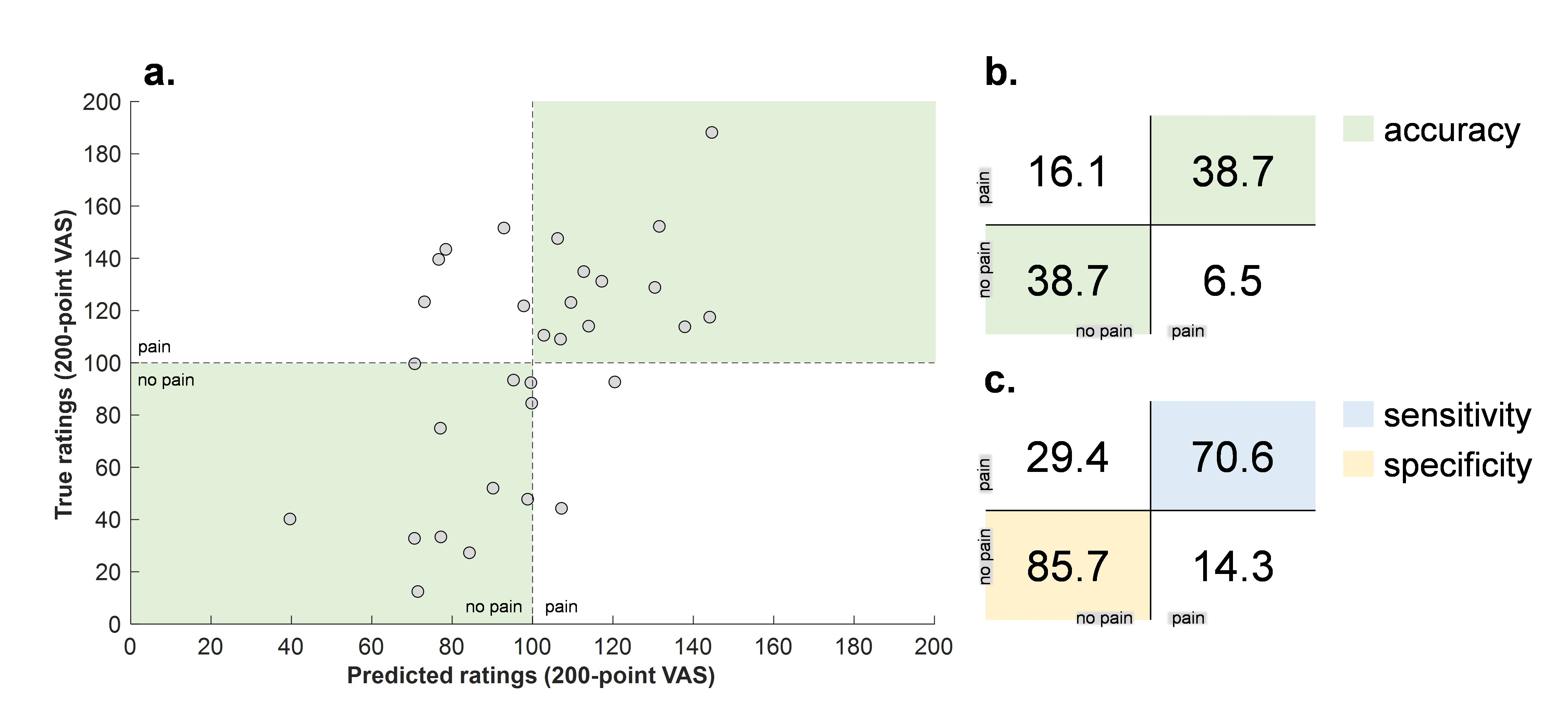

Supplement: Supplementary file 2 — High resolution image (TIF 1227 kb) [file 12021_2021_9527_MOESM1_ESM.tif]
