# Supplementary material for: Multiple Functional Brain Networks Related to Pain Perception Revealed by fMRI
Source: Neuroinformatics. 2021 Jun 8;20(1):155–72. doi: 10.1007/s12021-021-09527-6 (PMC9537130; doi:10.1007/s12021-021-09527-6)
Supplement: Supplementary file 3 — (PDF 73 kb) [file 12021_2021_9527_MOESM2_ESM.pdf]

Supplementary Table 1

Anatomical Descriptions for the Top 10% of Component 1 Loadings

Brain Regions (Harvard-Oxford Atlas)	Cluster Volume (mm^3)	Brodmann's Area for Peak Location	MNI Coordinates for Peak Locations			Component Loading
			x	y	z	
positive loadings						
cluster 1: bilateral	211356					
cerebellum (lobule VI)		37	21	-52	-22	0.3271
occipital fusiform gyrus / cerebellum crus 1		19	-45	-67	-19	0.3121
lateral occipital cortex, inferior division		19	-39	-79	-16	0.307
precentral gyrus		6	-42	-13	56	0.3031
precentral gyrus		4	-36	-19	62	0.2985
lateral occipital cortex, superior division		19	30	-73	26	0.295
occipital fusiform gyrus		19	45	-64	-19	0.2945
precuneous cortex		7	3	-58	62	0.2918
precuneous cortex		n/a	0	-52	65	0.2897
lingual gyrus		18	6	-70	-13	0.2876
lateral occipital cortex, superior division		7	27	-61	56	0.2862
lateral occipital cortex, superior division		7	24	-67	53	0.2858
postcentral gyrus		4	-51	-19	50	0.284
lingual gyrus		18	9	-73	-16	0.2837
precentral gyrus		6	-30	-7	65	0.2811
precentral gyrus		6	-27	-22	68	0.2782
occipital fusiform gyrus		19	36	-79	-16	0.2735
lateral occipital cortex, inferior division		19	33	-82	-13	0.2733
postcentral gyrus		4	0	-40	71	0.2719
lateral occipital cortex, inferior division		19	36	-82	2	0.2674
lateral occipital cortex, superior division		19	-30	-79	20	0.2651
lateral occipital cortex, superior division		7	-27	-61	53	0.2595
lingual gyrus		17	-6	-88	-4	0.2576
superior parietal lobule		7	-27	-55	59	0.2575
occipital fusiform gyrus		18	18	-88	-13	0.2572
intracalcarine cortex		17	12	-85	-1	0.257
occipital fusiform gyrus		18	-15	-85	-16	0.2543
lingual gyrus		17	-3	-85	-10	0.2516
lateral occipital cortex, superior division		7	-18	-64	56	0.2514
superior parietal lobule		n/a	-36	-40	59	0.2505
lingual gyrus		18	-12	-88	-13	0.2503
lingual gyrus		18	-9	-73	-19	0.2346
vermis		n/a	3	-73	-37	0.2092
juxtapositional lobule cortex (supplementary motor cortex)		6	-3	-1	71	0.2091
juxtapositional lobule cortex (supplementary motor cortex)		n/a	-3	-7	74	0.2068
cingulate gyrus, posterior division		n/a	0	-43	5	0.1791
cluster 2: left hemisphere	1107					
juxtapositional lobule cortex (supplementary motor cortex)		24	-3	2	47	0.2201
cluster 3: right hemisphere	864					
cerebellum (lobule VIII)		n/a	30	-46	-52	0.1984
cluster 4: right hemisphere	810					
middle frontal gyrus		6	33	2	62	0.2032

cluster 5: right hemisphere	378					
postcentral gyrus		4	54	-19	47	0.1922
cluster 6: right hemisphere	108					
occipital pole		n/a	3	-94	14	0.1802
cluster 7: left hemisphere	54					
thalamus		n/a	-12	-16	5	0.178
cluster 8: right hemisphere	27					
occipital pole		n/a	3	-91	29	0.1733
cluster 9: right hemisphere	27					
postcentral gyrus		2	48	-31	53	0.1734

Note. Negative loadings: no negative loadings passed threshold
