# Supplementary material for: Multiple Functional Brain Networks Related to Pain Perception Revealed by fMRI
Source: Neuroinformatics. 2021 Jun 8;20(1):155–72. doi: 10.1007/s12021-021-09527-6 (PMC9537130; doi:10.1007/s12021-021-09527-6)
Supplement: Supplementary file 4 — (PDF 99 kb) [file 12021_2021_9527_MOESM3_ESM.pdf]

Supplementary Table 2

Anatomical Descriptions for the Top 10% of Component 2 Loadings

Brain Regions (Harvard-Oxford Atlas)	Cluster Volume (mm^3)	Brodmann's Area for Peak Location	MNI Coordinates for Peak Locations			Component Loading
			x	y	z	
positive loadings						
cluster 1: right hemisphere	64017					
temporal pole		38	57	14	-7	0.2149
frontal pole		46	42	47	2	0.1894
central opercular cortex		22	63	-19	17	0.1745
frontal pole		45	39	47	17	0.1445
supramarginal gyrus, anterior division		2	57	-28	44	0.14
heschl's gurs (includes H1 and H2)		48	57	-10	5	0.1398
supramarginal gyrus, anterior division		40	54	-34	47	0.1365
middle frontal gyrus		n/a	48	8	44	0.1359
supramarginal gyrus, posterior division		40	51	-40	44	0.135
inferior frontal gyrus, pars opercularis		44	54	17	29	0.133
middle frontal gyrus		6	45	5	47	0.1323
putamen		48	33	-13	-4	0.1314
middle frontal gyrus		45	51	32	20	0.1281
insular cortex (posterior)		48	36	-13	11	0.1265
inferior frontal gyrus, pars opercularis		48	54	20	17	0.1253
insular cortex (posterior)		48	39	-10	-10	0.1179
putamen		34	30	-1	-7	0.1082
pallidum		n/a	27	-4	-1	0.106
lateral occipital cortex, superior division		7	36	-64	44	0.099
insular cortex (anterior)		48	33	5	5	0.0989
insular cortex (anterior)		47	33	26	-1	0.0955
cluster 2: bilateral	45765					
cerebellum (crus 1)		n/a	-30	-64	-31	0.1668
intracalcarine cortex		17	0	-76	8	0.154
cerebellum (crus 1)		n/a	-12	-73	-31	0.1345
precuneous cortex		7	-9	-70	38	0.1262
cerebellum (lobule VI)		37	-27	-43	-31	0.1239
n/a		n/a	-21	-40	-31	0.1222
cuneal cortex		18	3	-79	29	0.1199
lingual gyrus		18	-12	-58	-16	0.1151
cerebellum (lobule IV)		18	-9	-55	-13	0.1145
lingual gyrus		18	9	-70	35	0.1108
n/a		n/a	0	-49	-22	0.1097
precuneous cortex		7	12	-70	41	0.1088
intracalcarine cortex		18	12	-67	32	0.108
cerebellum (lobule VIII)		n/a	-24	-61	-40	0.1076
n/a		27	6	-34	-34	0.1024
lingual gyrus		18	-15	-55	-28	0.1016
cerebellum (lobule VIII)		n/a	-21	-67	-40	0.1015
n/a		n/a	-12	-58	-34	0.1015
vermis		n/a	0	-55	-34	0.0972
cluster 3: left hemisphere	39636					
temporal pole		n/a	-48	14	-10	0.17
inferior frontal gyrus, pars opercularis		48	-57	11	-1	0.1514
central opercular cortex		48	-57	2	2	0.151

parietal operculum cortex		42	-57	-31	20	0.1378
insular cortex (anterior)		48	-36	14	2	0.1315
supramarginal gyrus, anterior division		40	-57	-37	38	0.1253
middle temporal gyrus, temporooccipital part		21	-45	-46	47	0.1236
frontal orbital cortex		47	-33	26	-7	0.1234
n/a		37	-39	-49	44	0.1217
insular cortex (posterior)		48	-39	-10	-10	0.1211
angular gyrus		7	-33	-58	38	0.1209
supramarginal gyrus, posterior division		40	-36	-46	41	0.1192
inferior frontal gyrs		44	-45	11	29	0.109
insular cortex (posterior)		48	-33	-19	2	0.1056
cluster 4: bilateral	29781					
paracingulate gyrus		n/a	0	26	38	0.1846
superior frontal gyrus		6	3	14	59	0.1686
superior frontal gyrus		6	-15	11	59	0.0956
cluster 5: left hemisphere	13608					
frontal pole		47	-39	44	-1	0.1578
frontal pole		10	-30	53	8	0.1357
cluster 6: bilateral	13122					
n/a		n/a	12	2	8	0.149
n/a		n/a	-12	5	8	0.1426
thalamus		n/a	12	-10	5	0.13
n/a		n/a	-6	-19	-7	0.1
n/a		n/a	-9	-16	-4	0.0977
n/a		n/a	9	-16	-7	0.0973
cluster 7: right hemisphere	3726					
cerebellum (lobule VI)		n/a	30	-58	-31	0.1465
cluster 8: left hemisphere	2052					
cingulate gyrus, posterior division		n/a	-3	-28	23	0.1355
cluster 9: left hemisphere	1053					
middle frontal gyrus		8	-27	11	53	0.1066
middle frontal gyrus		6	-33	2	44	0.1016
cluster 10: right hemisphere	783					
postcentral gyrus		3	24	-37	65	0.1116
cluster 11: right hemisphere	297					
n/a		n/a	12	-58	-34	0.1045
cluster 12: left hemisphere	297					
precentral gyrus		48	-39	-13	32	0.1028
cluster 13: left hemisphere	135					
insular cortex (posterior)		48	-36	-16	14	0.1034
cluster 14: right hemisphere	135					
precentral gyrus		6	21	-25	56	0.0978
cluster 15: left hemisphere	108					
frontal orbital cortex		48	-24	8	-10	0.0987
cluster 16: right hemisphere	54					
n/a		n/a	9	-73	-31	0.0936
cluster 17: right hemisphere	54					
n/a		n/a	12	-37	-31	0.0942
cluster 18: right hemisphere	54					
insular cortex (anterior)		47	33	20	2	0.0972
cluster 19: right hemisphere	54					
precentral gyrus		n/a	39	-10	35	0.0986

Note. Negative loadings: no negative loadings passed threshold
