# Supplementary material for: Multiple Functional Brain Networks Related to Pain Perception Revealed by fMRI
Source: Neuroinformatics. 2021 Jun 8;20(1):155–72. doi: 10.1007/s12021-021-09527-6 (PMC9537130; doi:10.1007/s12021-021-09527-6)
Supplement: Supplementary file 5 — (PDF 108 kb) [file 12021_2021_9527_MOESM4_ESM.pdf]

Supplementary Table 3

Anatomical Descriptions for the Top 10% of Component 4 Loadings

Brain Regions (Harvard-Oxford Atlas)	Cluster Volume (mm^3)	Brodmann's Area for Peak Location	MNI Coordinates for Peak Locations			Component Loading
			x	y	z	
positive loadings						
cluster 1: right hemisphere	162					
frontal pole		46	48	47	-1	0.0693
negative loadings						
cluster 1: bilateral	102870					
cingulate gyrus, posterior division		23	-3	-55	26	-0.142
cingulate gyrus, posterior division		n/a	0	-46	23	-0.1409
cingulate gyrus, posterior division		27	-9	-43	2	-0.1242
cingulate gyrus, posterior division		n/a	-6	-46	5	-0.1236
precuneous cortex		n/a	0	-43	56	-0.1141
parahippocampal gyrus, anterior division		30	-24	-19	-19	-0.1132
postcentral gyrus		5	12	-43	62	-0.1115
precentral gyrus		n/a	-3	-31	50	-0.108
precentral gyrus		n/a	0	-31	62	-0.1076
superior parietal lobule		2	24	-40	59	-0.1033
cingulate gyrus, posterior division		27	12	-43	-1	-0.1023
parahippocampal gyrus, posterior division		30	-24	-37	-16	-0.0994
precentral gyrus		4	9	-31	71	-0.0976
precentral gyrus		4	-6	-28	71	-0.0968
precentral gyrus		n/a	0	-22	53	-0.0941
temporal fusiform cortex, posterior division		20	-30	-31	-22	-0.094
lingual gyrus		30	-18	-43	-13	-0.0936
postcentral gyrus		n/a	-12	-40	56	-0.0923
postcentral gyrus		3	36	-28	56	-0.0922
juxtapositional lobule cortex		n/a	3	-4	77	-0.0895
precentral gyrus		6	45	-10	56	-0.0887
precentral gyrus		6	6	-16	80	-0.0819
precentral gyrus		6	36	-13	65	-0.0816
precentral gyrus		6	21	-19	71	-0.0816
cingulate gyrus, posterior division		23	-3	-16	38	-0.0786
precuneous cortex		7	6	-76	38	-0.0774
temporal occipital fusiform cortex		37	-27	-52	-13	-0.0769
postcentral gyrus		4	39	-19	47	-0.0757
temporal fusiform cortex, posterior division		37	-42	-40	-28	-0.0751
cuneal cortex		18	12	-79	32	-0.0748
hippocampus		37	27	-31	-4	-0.073
postcentral gyrus		6	-24	-28	65	-0.0715
hippocampus		37	24	-34	-1	-0.0714
postcentral gyrus		4	-18	-31	65	-0.0705
cuneal cortex		18	-6	-79	26	-0.0694
superior frontal gyrus		6	-9	-1	71	-0.0691

cingulate gyrus, anterior division		24	0	-1	35	-0.0677
n/a		27	18	-31	-1	-0.0659
cluster 2: bilateral	41526					
frontal pole		10	3	59	2	-0.137
frontal pole		10	-15	62	20	-0.1002
frontal orbital cortex		47	-27	32	41	-0.0933
frontal pole		9	-21	50	29	-0.0907
frontal pole		9	-18	47	38	-0.0883
frontal pole		10	12	62	23	-0.0869
frontal pole		9	-15	44	41	-0.085
cluster 3: left hemisphere	39501					
lateral occipital cortex, superior division		39	-48	-64	23	-0.142
middle temporal gyrus, posterior division		20	-57	-10	-16	-0.1384
angular gyrus		37	-54	-58	14	-0.1301
middle temporal gyrus, anterior division		21	-57	-1	-25	-0.1167
superior temporal gyrus, posterior division		22	-60	-40	8	-0.1024
middle temporal gyrus, posterior division		21	-66	-19	-10	-0.0944
temporal pole		38	-51	17	-28	-0.0867
temporal pole		38	-54	17	-22	-0.0842
temporal pole		n/a	-39	2	-46	-0.0799
planum temporale		41	-45	-37	8	-0.0753
superior temporal gyrus, posterior division		21	-48	-40	5	-0.0728
frontal orbital cortex		38	-42	29	-19	-0.0727
planum temporale		22	-54	-25	2	-0.0707
temporal pole		38	-33	26	-28	-0.0705
planum temporale		48	-48	-25	2	-0.0703
cluster 4: right hemisphere	14931					
angular gyrus		39	45	-55	23	-0.121
supramarginal gyurs, posterior division		41	48	-37	11	-0.093
superior temporal gyrus, posterior division		21	51	-31	-1	-0.0747
heschle's gyrus (includes H1 and H2)		48	36	-25	14	-0.0702
cluster 5: right hemisphere	6912					
middle temporal gyrus, anterior division		21	60	-4	-19	-0.102
temporal gyrus		21	57	5	-25	-0.0855
temporal pole		21	54	5	-31	-0.0844
inferior temporal gyrus, anterior division		n/a	57	-4	-37	-0.0773
cluster 6: right hemisphere	2700					
parahippocampal gyrus, anterior division		20	27	-19	-19	-0.1039
temporal fusiform cortex, posterior division		20	30	-31	-22	-0.0761
temporal occipital fusiform cortex		37	42	-40	-28	-0.0703
temporal occipital fusiform cortex		37	42	-46	-25	-0.0699
cluster 7: right hemisphere	1539					
cerebellum (crus 2)		n/a	27	-76	-37	-0.0851
cluster 8: left hemisphere	972					
postcentral gyrus		4	-45	-13	32	-0.0768
postcentral gyrus		3	-54	-13	35	-0.0714
cluster 9: left hemisphere	783					
subcallosal cortex		25	-3	17	-13	-0.0832
cluster 10: right hemisphere	648					
frontal pole		9	21	38	47	-0.0777

superior frontal gyrus		9	21	29	41	-0.0701
cluster 11: right hemisphere	513					
cerebellum (lobule IX)		n/a	9	-49	-46	-0.0848
cluster 12: fight hemisphere	405					
temporal pole		38	33	26	-31	-0.0725
temporal pole		n/a	24	23	-34	-0.0702
cluster 13: left hemisphere	351					
cerebellum (crus 2)		n/a	-24	-76	-34	-0.0788
cluster 14: left hemisphere	216					
insular cortex		48	-36	-10	11	-0.0717
cluster 15: right hemisphere	108					
middle temporal gyrus, posterior division		21	69	-31	-7	-0.0693
cluster 16: left hemisphere	108					
parietal operculum cortex		48	-39	-31	17	-0.0678
cluster 17: right hemisphere	108					
postcentral gyrus		3	54	-10	32	-0.0661
cluster 18: left hemisphere	81					
cerebellum (lobule IX)		n/a	-6	-52	-46	-0.0693
cluster 19: right hemisphere	81					
frontal orbital cortex		47	39	32	-19	-0.0674
cluster 20: right hemisphere	81					
lingual gyrus		19	21	-58	-7	-0.0656
cluster 21: right hemisphere	81					
lateral occipital cortex, superior division		7	21	-70	41	-0.071
cluster 22: left hemisphere	27					
temporal pole		36	-30	2	-28	-0.0658
cluster 23: right hemisphere	27					
central opercular cortex		48	63	-4	11	-0.0649
