# Supplementary material for: Multiple Functional Brain Networks Related to Pain Perception Revealed by fMRI
Source: Neuroinformatics. 2021 Jun 8;20(1):155–72. doi: 10.1007/s12021-021-09527-6 (PMC9537130; doi:10.1007/s12021-021-09527-6)
Supplement: Supplementary file 6 — (PDF 30 kb) [file 12021_2021_9527_MOESM5_ESM.pdf]

Supplementary Table 4

Descriptive Statistics for Pain Rating and Network Activation Data, by Temperature Category

	High Temperatures		Low Temperatures	
Variable	*Mean*	*S.E.*	*Mean*	*S.E.*
Pain Rating	118.83	5.06	61.16	5.82
Component 1	-0.17	0.01	-0.22	0.01
Component 2	0.11	0.02	0.02	0.02
Component 4	0	0.02	-0.04	0.03

Note. Pain ratings are given on a 200-point VAS scale, Component activations are given by FIR-based predictor weights outputted by fMRI-CPCA (see section 2.6.). S.E. = standard error of the mean.
