# Supplementary material for: Multiple Functional Brain Networks Related to Pain Perception Revealed by fMRI
Source: Neuroinformatics. 2021 Jun 8;20(1):155–72. doi: 10.1007/s12021-021-09527-6 (PMC9537130; doi:10.1007/s12021-021-09527-6)
Supplement: Supplementary file 7 — (DOCX 25.6 kb) [file 12021_2021_9527_MOESM6_ESM.docx]

**Supplementary Materials for the following paper:**

Multiple Functional Brain Networks Related to Pain Perception Revealed by fMRI

Matteo Damascelli^1,6,7^, Todd S. Woodward^2,6^, Nicole Sanford^2,6^, Hafsa B. Zahid^2,6^, Ryan Lim^6^, Alexander Scott^3,5^, & John K. Kramer^4,7*^

^1^Department of Psychology, University of British Columbia, 2136 West Mall, Vancouver, BC V6T 1Z4, ^2^Department of Psychiatry, University of British Columbia, 2255 Wesbrook Mall, Vancouver, BC V6T 2A1, ^3^Department of Physical Therapy, University of British Columbia, 2177 Wesbrook Mall, Vancouver, BC V6T 1Z3, ^4^School of Kinesiology, University of British Columbia, 6081 University Blvd, Vancouver, BC V6T 1Z1, ^5^Centre for Hip Health and Mobility, Robert H. N. Ho Research Centre, 2635 Laurel St, Vancouver, BC V5Z 1M9, ^6^BC Mental Health & Addictions Research Institute, BC Children’s Hospital Research Institute, 938 West 28^th^ Ave, Vancouver, BC V5Z 4H4, ^7^ICORD, Blusson Spinal Cord Centre, 818 West 10th Ave, Vancouver, BC V5Z 1M9.

* denotes corresponding author, John K. Kramer (tel: 604–675–8876, email: [kramer@icord.org](mailto:kramer@icord.org))

Table of Contents

**Supplementary Methods3**

Pre-processing pipeline3

Reorientation3

Slice-time Correction3

Realignment3

Co-registration4

Segmentation5

Normalization55

Smoothing6

**References7**

**Supplementary Methods**

**Pre-processing Pipeline**

All pre-processing for this study was completed in SPM 12, within MATLAB 2014a. Each step and algorithm will be explained in the sections that follow. SPM 12 is available from <https://www.fil.ion.ucl.ac.uk/spm/software/spm12/>.

**Reorientation.** Reorientation was performed manually on both structural and functional scans for each participant. Images were displayed in the SPM 12 GUI within MATLAB 2014a. The origin was placed on the anterior commissure, and the scans reoriented such that the anterior commissure – posterior commissure plane assumed a horizontal position.

**Slice-time Correction.** Due to the temporal lag that occurs during the acquisition of a functional 3D image using fMRI (i.e. a single scan is collected by taking 2-D snapshots of the brain in a particular order, until the entire brain has been covered, a sequence which usually takes around TR = 2-3 seconds), it was necessary to perform slice-time correction. Slice-time correction involves shifting signal phase by a particular amount such that the data collected on each 2-D slice correspond to an equivalent moment in time. This correction is applied to each functional scan taken for each participant. The amount of signal shifting is determined by choosing a reference slice in the TR sequence. We chose slice 21 of each scan as a reference point for signal shifting, given that 42 slices were acquired, and middle slices are generally good practice. SPM 12 applies a *sinc* interpolation algorithm by default to accomplish necessary shifting of other slices in the sequence to align with the reference slice (Sladky et al., 2011). This algorithm compensates for interpolation and “wrap-around” effects (Calhoun et al., 2000; Sladky et al., 2011).

**Realignment.** SPM realignment algorithms were applied to functional scans to ensure that each voxel represented the same location in the brain throughout all participant runs; this is a fundamental assumption that must be met in order to perform further statistical analysis on the data. Any kind of movement while in the scanner will violate this assumption and must be corrected for. Thus, SPM utilizes algorithms designed to estimate the amount of motion, and correct for that motion, in each set of scans for each participant. Computationally, this is accomplished by first registering all image volumes in a time-series to a reference volume using a rigid-body transformation with three translational and three rotational parameters. Parameters for the closest match (between the reference volume and volume-to-be-matched) are determined by a minimizing a cost function, in this case the sum of squared differences between voxel intensities of the two images. After registration, the realignment parameters are applied to the corresponding original images to estimate the “true” images absent the head motion that occurred during scanning, a process referred to as spatial interpolation (WikiBooks, 2020). These two steps correspond to the “Estimate and Re-slice” option in the SPM 12 GUI. Estimate and Re-slice were implemented with default values for each sub-parameter option (e.g. Estimate includes parameters like “quality” and “separation” to be specified by the user), except for “Resliced Images”, under Re-slice options, in which we selected *Mean Image Only.

Realignment is generally accurate within a range of 1-3 mm translation in the x, y, and z coordinate directions, as well as 5 degrees of rotation in pitch, yaw or roll. In this study, we chose to exclude runs if they exceeded 4.5 mm of movement in x, y or z. Specific runs excluded were subject 10, run 5 and 6; subject 2, run 5; subject 4, run 1, 4 and 5. Exclusions based on rotational movement were not made as all runs were within the rotational threshold of 5 degrees.

**Co-registration.** Co-registration aligns functional images with structural images for each participant, to ensure that voxels in a structural image correspond to the same part of the brain as voxels in the functional images. We accomplished this in SPM by aligning structural image files with a “mean functional image” file calculated from all functional scans for a participant. Co-registration again uses rigid transformations to align the source image (the mean functional image) to the reference image (a subject’s structural image). In contrast to the registration step that occurs during realignment (see section “Realignment” above), the two images being registered are of different modalities (i.e. structural vs. functional, captured in the scanner with different T weightings). Thus, the three translational and rotational parameters are calculated by optimizing a different cost function called “mutual information” (Hermans, 2016).

**Segmentation.** Segmentation divides participant structural images (high-resolution T1 spoiled gradient recall images) into different tissue classes: grey matter (GM), white matter (WM), cerebro-spinal fluid (CSF), meninges and skull components. SPM uses a Gaussian Mixture Model that incorporates tissue probability maps (describing the probability that each voxel contains a certain type of tissue) to arrive at a robust solution. In SPM, the segmentation step also includes a step for correcting intensity non-uniformity; tissue partitions derived based on uncorrected images can be confounded by smooth intensity variations caused by RF inhomogeneities from the head coil of the MRI machine.

In the SMP 12 GUI, segmentation steps were implemented with default values, except for the following: under Save Bias Corrected, we selected Save Bias Corrected; for bias regularization, we selected light regularisation; for bias FWHM, we selected 60mm cut-off; for the first two tissue types, we set the number of Gaussians to 2; for tissue types 3, 4, 5, 6 we set number of Gaussians to 2, 3, 5, 2, respectively; under Native Tissue, we selected Native Space + Dartel Imported for tissues 1-3, default values were chosen for tissues 4-5, and none were selected for tissue 6; Warped Tissue was left to default value None for all tissues; under Warping & MRF, we selected Don’t do Cleanup for the Cleanup option, and Forward for Deformation Fields.

**Normalization.** Normalization warps subject images onto a standard template space, minimizing differences between individual brains and ensuring that activations can be compared, and statistics calculated at the group level. We implemented normalization through the “indirect normalization” option in SPM 12, which normalizes high-resolution structural images to a T1 template in MNI coordinate space. The normalization computes “deformation fields”, or warping parameters for the transformation of structural images into MNI space, and applies them to the functional scans to achieve equivalent transformations (Hermans, 2016). In the Writing Options section, we selected a voxel size of 3×3×3 mm.

**Smoothing.** Smoothing is a typical last step in preprocessing; it averages the signal in every voxel with a weighted sum of its neighbours. The weighting is specified by a Gaussian kernel, whose size is given by its Full Width at Half Maximum (FWHM). Spatial smoothing increases the signal to noise ratio by suppressing the noise and effects produced by residual inter-subject variability after normalization and it improves validity of experimental tests in voxel-wise analysis by allowing application of Gaussian random field theory during statistical analysis. Smoothing can also spread activations across sulci (depressions or fissures on the surface of the brain) and increase partial-volume effects (averaging across different tissues) by changing all the data points into Gaussian distributions. In this study, we chose a kernel of size 6×6×6.

**References**

Calhoun, V., Golay, X., & Pearlson, G. (2000). Improved fMRI slice timing correction: interpolation errors and wrap around effects. In *Proceedings, ISMRM, 9th annual meeting, Denver* (p. 810).

Hermans, E. (2016). SPM 12 Starters’ Guide. Retrieved from: <https://www.ernohermans.com/wp-content/uploads/2016/09/spm12_startersguide.pdf>.

Sladky, R., Friston, K. J., Tröstl, J., Cunnington, R., Moser, E., & Windischberger, C. (2011). Slice-timing effects and their correction in functional MRI. *Neuroimage*, *58*(2), 588-594.

WikiBooks (2020). Neuroimaging Data Processing/Realignment. Retrieved from: <https://en.wikibooks.org/wiki/Neuroimaging_Data_Processing/Realignment#SPM>.
